# Advanced Constitutive Modeling of the Thixotropic Elasto-Visco-Plastic Behavior of Blood: Steady-State Blood Flow in Microtubes

**DOI:** 10.3390/ma14020367

**Published:** 2021-01-13

**Authors:** Konstantinos Giannokostas, Yannis Dimakopoulos, Andreas Anayiotos, John Tsamopoulos

**Affiliations:** 1 Laboratory of Fluid Mechanics and Rheology, Department of Chemical Engineering, University of Patras, 26504 Patras, Greece; giannoko@upatras.gr (K.G.); tsamo@chemeng.upatras.gr (J.T.); 2 Department of Mechanical and Materials Engineering, Cyprus University of Technology, Limassol 3036, Cyprus; andreas.anayiotos@cut.ac.cy

**Keywords:** blood flow, blood thixotropy, blood viscoelasticity, aggregation, rouleaux, hemodynamics, microtubes, relaxation time, CFL, Fåhraeus effect, plasma viscoelasticity, wall shear & normal stresses, interfacial shear & normal stresses, personalized hemorheology

## Abstract

The present work focuses on the in-silico investigation of the steady-state blood flow in straight microtubes, incorporating advanced constitutive modeling for human blood and blood plasma. The blood constitutive model accounts for the interplay between thixotropy and elasto-visco-plasticity via a scalar variable that describes the level of the local blood structure at any instance. The constitutive model is enhanced by the non-Newtonian modeling of the plasma phase, which features bulk viscoelasticity. Incorporating microcirculation phenomena such as the cell-free layer (CFL) formation or the Fåhraeus and the Fåhraeus-Lindqvist effects is an indispensable part of the blood flow investigation. The coupling between them and the momentum balance is achieved through correlations based on experimental observations. Notably, we propose a new simplified form for the dependence of the apparent viscosity on the hematocrit that predicts the CFL thickness correctly. Our investigation focuses on the impact of the microtube diameter and the pressure-gradient on velocity profiles, normal and shear viscoelastic stresses, and thixotropic properties. We demonstrate the microstructural configuration of blood in steady-state conditions, revealing that blood is highly aggregated in narrow tubes, promoting a flat velocity profile. Additionally, the proper accounting of the CFL thickness shows that for narrow microtubes, the reduction of discharged hematocrit is significant, which in some cases is up to 70%. At high pressure-gradients, the plasmatic proteins in both regions are extended in the flow direction, developing large axial normal stresses, which are more significant in the core region. We also provide normal stress predictions at both the blood/plasma interface (INS) and the tube wall (WNS), which are difficult to measure experimentally. Both decrease with the tube radius; however, they exhibit significant differences in magnitude and type of variation. INS varies linearly from 4.5 to 2 Pa, while WNS exhibits an exponential decrease taking values from 50 mPa to zero.

## 1. Introduction

Understanding blood flow is of high theoretical and practical importance as it is directly associated with the pathophysiology and the development of diseases such as endotheliitis, microangiopathy, COVID-19 [1,2] in the microvasculature of any human being. Consequently, a consistent dynamic model is necessary for assessing the hemodynamic resistance and its regulation in the microcirculation [3]. We have already developed an integrated constitutive model of blood rheology in our recent work [4]. Blood has a pronounced non-Newtonian character, primarily attributed to aggregation, disaggregation, deformation, orientation, and migration of the erythrocytes [5,6,7,8,9]. Our model encompassed all the crucial aspects of blood rheology, including yield stress [10,11], thixotropic effects [12,13,14], viscous dissipation, and elasticity, which are associated with the aggregation/disaggregation of the erythrocytes [15,16]. The latter features are accompanied by the introduction of a microstructural state indicator that dynamically responds to the stresses currently present in the system. Our model was fitted to existing steady and transient experiments and predicted basic rheometric flows such as intermittent shear steps, LAOS, triangular shear step, and uniaxial flow. However, a consistent constitutive stress model for blood is insufficient to adequately describe blood flow in microtubes where hemodynamical phenomena occur [3].

Turning from rheometric to even one-dimensional flows gives rise to some of the prominent phenomena in the microscale that can be observed both in vitro and in vivo. These are the Fåhraeus–Lindqvist [17] and Fåhraeus [18] effects. The Fähraeus effect accounts for the reduction of the bulk hematocrit, whereas the Fähraeus-Lindqvist effect for the decrease in the apparent viscosity. They are interrelated and caused by the cross-stream migration of RBCs in tube flow, leading to a two-phase configuration [19] consisting of an RBC-rich central region and a cell-depleted annular layer adjacent to the microtube wall [3,20,21,22]. The latter region is interchangeably called the Cell-Free Layer (CFL) or the Cell-Depleted Layer (CDL) [5], and it is a well-known hemodynamic feature in microcirculation. The lateral migration of RBCs in microcirculation is governed by cell-wall hydrodynamic interactions, which drive the cells away from the wall, and by cell-cell hydrodynamic interactions, which tend to disperse back the RBCs [23] from low shear regions to high shear regions. This is not exclusively a blood flow phenomenon, but it is also observed in polymeric solutions, where it is called stress-gradient induced migration [24] or in the electro-osmotic transfer of polymeric chains [25].

The CFL thickness depends on the tube diameter [26], the concentration of RBCs (the systemic hematocrit, which ranges widely with the age and the sex from 35% to 45%) [27], and the flow rate [28]. When the CFL thickness increases, the apparent blood viscosity decreases [3]. RBC aggregation can enhance the lateral migration, increasing the CFL thickness, although increased cell-to-cell interactions (due to increased cell packing) can counter this effect to some extent. Also, CFL plays a pivotal role in the microvascular network as a lubricating layer since it reduces the friction between the RBC core and the vessel wall, as observed both in vivo [29] and in vitro [21,30] flows. The CFL has another prominent role since it also acts as a barrier between the tube and vascular beds, thus it also supports biochemical processes. For example, a wider CFL increases the Nitric Oxide (NO) diffusion path to be scavenged by hemoglobin in the RBCs. It also affects oxygen delivery from the RBCs to the tissue [31,32]. Consequently, an accurate determination of the thickness of the plasma layer near the wall is necessary for a blood flow study, especially when the examined range of this cross-sectional distance is similar to the arterioles size.

Regarding the effect of RBCs aggregability on the axial velocity profile [33,34], most of the basic investigations have conducted experiments in straight tubes or performed measurements in arterioles, highlighting the bluntness of the velocity profiles [35]. It has been shown that aggregation factors assist the migration of RBCs, causing the development of blunt velocity profiles and increased viscosity in the core flow region [22,36,37,38,39,40]. The latter phenomenon is more pronounced in low shear-rates, as it was stated by Cokelet and Goldsmith [21], who quantified the interplay between aggregation and hydrodynamic resistance to flow. Similar conclusions have been drawn by Sherwood et al. [41]. They studied the spatial variation of the CFL in bifurcating microchannels in conjunction with the aggregation effect on the local velocity configuration experimentally.

The in vivo data are limited [33,34,40]. All relative works conclude that the correlation between hematocrit, RBC agglomeration, and CFL formation is much more complicated. In the vasculature, vessels exhibit multiple bifurcations, leading to non-uniform velocity and cell distributions, and consequently, the aggregation effects in more complex geometries require further elucidation [42].

Previous mathematical models [26,43] treated both fluid phases as generalized Newtonian fluids, which is a quite simplified approach for blood flows in microtubes. Das et al. [44] proposed a two-phase Casson model to describe the in vitro velocity profiles of blood flow in low flow rates, while Sriram et al. [27] adopted the well-known Quemada model [45] for the determination of the CFL thickness under various rheological conditions. More recently, Moyers-Gonzalez and Owens [46] used principles of the kinetic theory to derive a non-homogeneous hemorheological model, which was applied for the evaluation of the CFL thickness using empirical laws for the reduction of the hematocrit within the microtube. The model was adopted for the first time in multidimensional flows by Dimakopoulos et al. [47]. Another approach is through multi-particle flow models [48,49,50,51,52], which can also give accurate predictions of local variation of the CFL width, with the major drawback being the high computational cost. Additionally, Qi et al. [53] and Narshimhan et al. [54] based on a coarse-grained theory predicted migration effects and the erythrocytes concentration along with the CFL layer in Couette flow and pressure-driven flow in tubes. This model takes into consideration the wall-induced hydrodynamic lift and the cell-cell interactions, predicting the Fähraeus-Lindqvist effect.

In this work, we combine both hemorheological features in a comprehensive mathematical formulation for the two-phase blood flow in microtubes. We invoke a thixotropic elasto-visco-plastic (TEVP) constitutive model to accurately predict the stresses in the blood in the RBC-rich central region. This model is described in detail in our previous work [4] and fitted on the steady and transient experimental data of McMillan et al. [55]. Following experimental evidence, the plasma phase is treated as a viscoelastic fluid, represented by the linear form of the well-known Phan-Tien-Tanner (or PTT) model. Through our advanced modelling, we try to address some of the open questions in microcirculation regarding the various migration mechanisms and the complex microstructure of blood. Particularly, we investigate the interplay between aggregation and the formation of the cell-free layer and the effect of aggregability on velocity profiles in microtubes with cross-sections comparable to the RBC diameter. Moreover, we examine the intensity of the migration and how it is affected by the diameter of the tube or the imposed pressure gradient. Additionally, we examine the distribution of the relaxation times of blood within the tube along with the aggregation size, which varies with the available space. Finally, we predict the normal and shear viscoelastic stresses in the flow field and how the rheological conditions affect the thixotropic microstructural state of blood.

Our contribution is divided into four sections: In Section 2, we briefly describe the problem formulation, the hemodynamic constraints, and the Thixotropic Elasto-Visco-Plastic (TEVP) constitutive modeling of the whole blood and the viscoelastic modeling of the plasma phase. Section 3 presents the validation of our model with experimental data. A thorough parametric analysis follows in Section 4, which describes the effect of the rheological parameters on blood behavior extensively.

## 2. Problem Formulation

**Figure 1:**
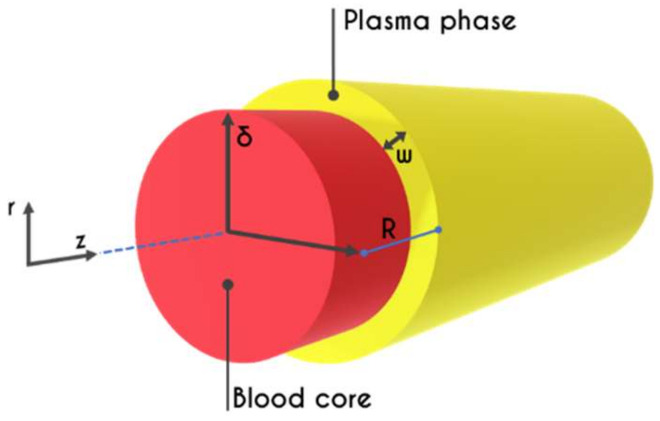
Schematic representation of a microtube of a radius R. It consists of a central RBC-rich region of radius δ, and an annular layer full of plasma and adjacent to the tube wall of thickness w=R−δ.

We consider the transient blood flow in two separate regions (i = 1,2), one for the RBC rich core (i = 1), placed at the center of the microtube, and a second one for the plasma layer, placed adjacent to the microtube’s wall (i = 2) (Figure 1). The momentum balance for the two-phase blood/plasma flow of density $\rho^{\left(i\right)}$ in a microtube of radius R is then written as

(1) $$\rho^{\left(i\right)}\left(\frac{\partial {\underline{U}}^{\left(i\right)}}{\partial t}\;+{\underline{U}}^{\left(i\right)}\cdot \underline{\nabla }\;{\underline{U}}^{\left(i\right)}\;\right)\;=\;\underline{\nabla }\cdot {\underline{\underline{\sigma }}}^{\left(i\right)}$$

where ${\underline{U}}^{\left(i\right)}$ is the velocity vector, ${\underline{\underline{\sigma }}}^{\left(i\right)}$ is the total stress tensor divided into an isotropic pressure and an extra stress as ${\underline{\underline{\sigma }}}^{\left(i\right)}=\;-p^{\left(i\right)}\underline{\underline{I}}+{\underline{\underline{\tau }}}^{\left(i\right)}$ with $\underline{\underline{I}}$ being the identity matrix.

Due to the different composition of each layer, we assume a different constitutive model for each phase. For the blood core, its modeling is based on the formulation reported in our tensorial TEVP form [4] and especially for the blood constitutive modeling. In this recent work, we invoked a consistent and validated model for TEVP materials proposed by Varchanis et al. [56], which features a coupling of the tensorial constitutive model by Saramito [57] for EVP materials with thixotropy. Another form of the proposed TEVP formulation, without thixotropy, was recently used to evaluate the transition between the solid and liquid state of elasto-viscoplastic fluids under extensional flow [58]. In addition, we assume a viscoelastic behavior for the proteinic plasma phase (CFL), which is mostly determined by fibrinogen concentration. The starting point is the decomposition of the extra stress tensor ${\underset{=}{\tau }}^{\left(i\right)}$ to solvent and viscoelastic terms as

(2) $${\underline{\underline{\tau }}}^{\left(i\right)}={{\underline{\underline{\tau }}}_{n}}^{\left(i\right)}+\;{\underline{\underline{\tau }}}_{\mathrm{ve}}{}^{\left(i\right)}$$

Regarding the modeling of the RBC-rich region, our model accounts for the viscoelastic contribution of the RBCs and the viscoelasticity of blood plasma since it has been fitted to high-shear rate plateau [4]. Consequently, there is no explicit Newtonian contribution $\left(\;{\underline{\underline{\tau }}}_{n}{}^{\left(1\right)}=0\right)$ as long as the solvent (plasma) is included in the viscoelastic term (${\underline{\underline{\tau }}}_{\mathrm{ve}}{}^{\left(1\right)}\;$). Additionally, the presence of plasma proteins [59,60] dominate the stress contribution in the pure plasma phase (CFL), and hence the Newtonian contribution (water) is assumed to be negligible there as well ${\underline{\underline{\tau }}}_{n}{}^{\left(2\right)}=0$.

### 2.1. Whole Blood Constitutive Modeling

The total rate of deformation tensor is decomposed into an elastic contribution ${\underline{\underline{D}}}_{e}{}^{\left(1\right)}$ and a viscoplastic one ${\underline{\underline{D}}}_{\mathrm{vp}}{}^{\left(1\right)}$ so that

(3) $${\underline{\underline{D}}}^{\left(1\right)}=\;{\underline{\underline{D}}}_{e}{}^{\left(1\right)}\;+{\underline{\underline{D}}}_{\mathrm{vp}}{}^{\left(1\right)}\;$$

The elastic term of the total deformation rate tensor ${\underline{\underline{D}}}_{e}{}^{\left(1\right)}$ accounts for memory effects and can be written as

(4) $${\underline{\underline{D}}}_{e}{}^{\left(1\right)}\;=\;\frac{1}{2\;G}\;\overset{\nabla }{{\underline{\underline{\tau }}}_{\mathrm{ve}}^{\left(1\right)}}\;$$

where the upper-convected time derivative is $\overset{\nabla }{{\underline{\underline{\tau }}}_{\mathrm{ve}}^{\left(1\right)}}=\frac{\partial {\underline{\underline{\tau }}}_{\mathrm{ve}}^{\left(1\right)}}{\partial t}+{\underline{U}}^{\left(1\right)}\cdot \underline{\nabla }\;{\underline{\underline{\tau }}}_{\mathrm{ve}}^{\left(1\right)}-\;{\left(\underline{\nabla }\;{\underline{U}}^{\left(1\right)}\right)}^{T}\cdot {\underline{\underline{\tau }}}_{\mathrm{ve}}^{\left(1\right)}-{\underline{\underline{\tau }}}_{\mathrm{ve}}^{\left(1\right)}\cdot \left(\underline{\nabla }\;{\underline{U}}^{\left(1\right)}\right)$, while G denotes the elastic modulus of blood. The effect of plasticity is introduced via a multiplication of specific functions and contributions given by the expression

(5) $${\underline{\underline{D}}}_{\mathrm{vp}}{}^{\left(1\right)}=\;f\left[\mathrm{tr}\left({\underline{\underline{\tau }}}_{\mathrm{ve}}^{\left(1\right)}\right)\right]\max\left(0,\frac{\left|{\underline{\underline{\tau }}}_{\mathrm{ve}}^{\left(1\right)}\right|-{\;\tau }_{y}}{2{\;\eta }_{t}\;\left|{\underline{\underline{\tau }}}_{\mathrm{ve}}^{\left(1\right)}\right|}\right){\underline{\underline{\tau }}}_{\mathrm{ve}}^{\left(1\right)}\;$$

where $f\left[\mathrm{tr}\left({\underline{\underline{\tau }}}_{\mathrm{ve}}^{\left(1\right)}\right)\right]$ is a stress-related l-PTT function, $\left|{\underline{\underline{\tau }}}_{\mathrm{ve}}^{\left(1\right)}\right|$ is the magnitude of the stress tensor, $\eta_{t}$ is the plastic viscosity, and $\tau_{y}$ is the blood yield stress (see [4]). The l-PTT function is defined as

(6) $$f\left[\mathrm{tr}\left({\underline{\underline{\tau }}}_{\mathrm{ve}}^{\left(1\right)}\right)\right]=\;1\;+{\;\varepsilon }_{\mathrm{PTT}}\;\frac{\mathrm{tr}\left({\underline{\underline{\tau }}}_{\mathrm{ve}}^{\left(1\right)}\right)}{G}\;$$

where $\varepsilon_{\mathrm{PTT}}$ is a fitted parameter, introducing shear-thinning along with bounding of the extensional viscosity. Given that the total rate of deformation tensor is equal to ${\underline{\underline{D}}}^{\left(1\right)}=\;\frac{1}{2}\;\left[\underline{\nabla }{\underline{U}}^{\left(1\right)}+{\left(\underline{\nabla }{\underline{U}}^{\left(1\right)}\right)}^{T}\right]$ and combining Equations (3)–(6), we get the final form of the constitutive equation

(7) $$\overset{\nabla }{{\underline{\underline{\tau }}}_{\mathrm{ve}}^{\left(1\right)}}+2\;G\;f\left[\mathrm{tr}\left({\underline{\underline{\tau }}}_{\mathrm{ve}}^{\left(1\right)}\right)\right]\max\left(0,\frac{\left|{\underline{\underline{\tau }}}_{\mathrm{ve}}^{\left(1\right)}\right|-{\;\tau }_{y}}{2{\;\eta }_{t}\;\left|{\underline{\underline{\tau }}}_{\mathrm{ve}}^{\left(1\right)}\right|}\right){\underline{\underline{\tau }}}_{\mathrm{ve}}^{\left(1\right)}=2G\;{\underline{\underline{D}}}^{\left(1\right)}\;$$

The time evolution of the structure (or rouleaux) parameter is then given by Wei et al. [61] as

(8) $$\frac{d\lambda }{\mathrm{dt}}\;=\;\left(k_{1}+k_{2}\varphi^{n_{1}}\right)\left(1-\lambda \right)\;-{\;k}_{3}\varphi^{n_{2}}\lambda^{n_{3}}$$

The first term in the RHS of Equation (8) represents the rebuilding of rouleaux, while the second one refers to the disintegration process. We base the dependency of the level of structure explicitly on the stresses, via the parameter φ [61]

(9) $$\varphi =\;\max\left(0,\left|{\underline{\underline{\tau }}}_{\mathrm{ve}}^{\left(1\right)}\right|-{\;\tau }_{y}\right)$$

Yielding occurs when φ>0, and this is the von Mises criterion. The plastic viscosity of blood $\eta_{t}\left(\lambda \right)$ is a thixotropy-dependent variable via the structural variable λ

(10) $$\eta_{t}\left(\lambda \right)=\eta_{0}\lambda^{m_{1}}\;$$

where $\eta_{0}$,${\;m}_{1}$ are fitting parameters. Similarly, we can define the relaxation time of blood, χ, as the ratio of the plastic viscosity to the shear modulus as

(11) $$\chi \left(\lambda \right)=\chi_{0}\lambda^{m_{1}}\;,{\;\chi }_{0}=\;\frac{\eta_{0}}{G}$$

To extract realistic values for the set of the parameters of the constitutive model, we adopt a non-linear regression procedure [62] on experimental data for steady-state and transient experiments. The steady-state experiment is the simple shear flow one providing the shear stress response as a function of the imposed shear rate. Our model is fitted on the experimental data of McMillan et al. [55] for a hematocrit equal to 45%, and the set of the adjustable parameters (eleven in number) are reported in Table 1, along with the nomenclature.

Alternatively, a modified form of the Casson model [63] is commonly used to simulate blood behavior under steady-conditions. It encompasses the property of the yield stress along with the shear-thinning description of blood and is given by

(12) $$\sqrt{\tau_{\mathrm{rz}}^{\left(1\right)}({\dot{\gamma }}_{\mathrm{rz}})}=\sqrt{\tau_{y}}+\;\sqrt{\mu }\sqrt{{\dot{\gamma }}_{\mathrm{rz}}^{\left(1\right)}}\;$$

where $\tau_{y}$ and μ are the yield stress and the viscosity, respectively, while they both are fitted to the steady state experimental data of McMillan et al. [55] and tabulated in Table 2. A generalized Newtonian constitutive model, such as the Casson model [63], needs only steady state experiments so as for the adjustable parameters to be determined. A plethora of steady shear experiments with different hematocrits reported in the literature, enables the extraction of a mathematical correlation between core and discharged hematocrit.

### 2.2. Plasma Constitutive Modeling

Although in most investigations, blood plasma has been considered a Newtonian fluid, recent shear and extension dominated flow experiments prepared by Brust et al. [59] revealed that blood plasma features bulk viscoelasticity. They reported phenomena that have been widely studied, both experimentally and theoretically, in polymeric solutions [64,65,66], clearly indicating that human blood plasma has viscoelastic properties. In contrast to Newtonian fluids, blood plasma exhibited a shear-thinning viscosity. Despite the evidence of the existence of blood plasma viscoelasticity, only a few investigations take this into account, such as the work of Varchanis et al. [60], who provided a complete data set of the rheometric material functions of plasma rheology in simple shear and elongational flows. Finally, combining their results with previous in vivo measurements, they additionally found that the viscoelasticity of human blood plasma must not be ignored when examining the flow of whole blood in micro-tube, such as arterioles or capillaries. Consequently, we adopt viscoelastic constitutive modelling via the Phan-Thien-Tanner (or PTT) model [67,68,69]. The model is an extension of the Maxwell model accompanied with the upper convected derivative to include a function dependent upon $\mathrm{tr}({\underline{\underline{\tau }}}_{\mathrm{ve}}^{\left(2\right)})$, the trace of the viscoelastic stress tensor due to plasma proteins. The tensorial form in steady state and fully developed flow is then given by

(13) $$\frac{\partial {\underline{\underline{\tau }}}_{\mathrm{ve}}^{\left(2\right)}}{\partial t}+\left(\frac{1}{\lambda_{\mathrm{pl}}}+\frac{\varepsilon_{\mathrm{PTT},\mathrm{pl}}}{\eta_{\mathrm{pl}}}\mathrm{tr}({\underline{\underline{\tau }}}_{\mathrm{ve}}^{\left(2\right)})\right)\;{\underline{\underline{\tau }}}_{\mathrm{ve}}^{\left(2\right)}+\left[{\left(\underline{\nabla }{\;\underline{U}}^{\left(2\right)}\right)}^{T}\cdot {\underline{\underline{\tau }}}_{\mathrm{ve}}^{\left(2\right)}-{\underline{\underline{\tau }}}_{\mathrm{ve}}^{\left(2\right)}\cdot \underline{\nabla }{\underline{U}}^{\left(2\right)}\right]=2\frac{\eta_{\mathrm{pl}}}{\lambda_{\mathrm{pl}}}\;{\underline{\underline{D}}}^{\left(2\right)}\;$$

where $\lambda_{\mathrm{pl}}$ is the relaxation time of plasma and $\eta_{\mathrm{pl}}$ is the viscosity of plasma, which are both adjustable parameters while $\varepsilon_{\mathrm{PTT},\mathrm{pl}}$ is the PTT parameter which is responsible for bounding the extensional viscosity. Fitting the l-PTT formula to the data reported in [60], we extract the parameters of the model illustrated in Table 3. The one-dimensional forms of the above equations are presented in detail in Appendix A.

### 2.3. Hemodynamical Constraints

The model presented above is not complete, and its equations cannot be solved unless the location of the core/plasma interface or the core radius δ, can be determined. Hence, this location must be computed along with all the other unknowns, making this a moving boundary problem. To calculate it, we need to estimate the impact of the hemorheological parameters on the velocity and stress fields by introducing hemodynamical constraints, which quantify major microcirculation effects [70]. As such, we refer to the Fähraeus [71] and Fähraeus-Lindqvist [17] phenomena. Both are related to the increase of the CFL thickness as the tube radius decreases, attributed to the increasing tendency of the RBCs to migrate towards the center of the vessel [72]. Unfortunately, neither effect can be predicted using first principles, so we have to rely on experiments and develop suitable correlations. The Fähraeus effect is the reduction of the bulk hematocrit, whereas the Fähraeus-Lindqvist effect accounts for the decrease in the apparent viscosity ($\eta_{\mathrm{app}}$).

Before presenting these correlations, we need to clarify the different existing definitions of the hematocrit, which represents the volume fraction of RBCs in whole blood expressed as a percentage. Namely, these are the discharged hematocrit $H_{d}$, the tube hematocrit, $H_{t}$, and the core hematocrit, $H_{c}$. The discharged hematocrit is the velocity-weighted average of the local volume fraction of the erythrocytes given by

(14) $$H_{d}=\frac{\int_{0}^{R}H\left(r\right){\;U}_{z}\left(r\right)\;r\;\mathrm{dr}}{\int_{0}^{R}U_{z}\left(r\right)\;r\;\mathrm{dr}}$$

where $H\left(r\right)$ is the local volume fraction of RBCs across a section of the tube. Discharged hematocrit is a measure of its bulk value accounting for the fact that the local volume fraction at a specific radial position is carried by the local axial velocity, which also varies radially. Since the higher RBC concentration is near the tube center due to cell aggregation and it is carried by the higher local velocity, $H_{d}$ is larger than $H_{t}$, which is defined next. The tube hematocrit, $H_{t}$ represents the average hematocrit within the vessel, assuming that the velocity has a plug-flow (radially constant) profile. Hence, $H_{t}$ is given by

(15) $$H_{t}=\frac{\int_{0}^{R}H\left(r\right)\;r\;\mathrm{dr}}{\int_{0}^{R}\;r\;\mathrm{dr}}$$

In general and in this work, given that the RBC migration away from the wall leads to a considerable decrease of the local viscosity, which cannot be described due to the absence of any realistic differential model, we assume that $H\left(r\right)$ follows a simple distribution

(16) Hr=Hcr ∈ 0,δ 0r ∈ δ, R

which assigns a constant value to the hematocrit in the core region denoted by $H_{c}$ and a zero value in the CFL. If we substitute Equation (16) into Equation (15), we readily get a linear relationship between $H_{t}$ and $H_{c}$ given by

(17) $$H_{c}=H_{t}\;{\left(\frac{R}{\delta }\right)}^{2}$$

where δ is the core radius shown in Figure 1. Similarly, $H_{d}$ can be related to $H_{c}$ using a simple mass balance of RBCs in any cross-section

(18) $$H_{d}\;\left[\overset{\delta }{\underset{0}{\int }}U_{z}^{\left(1\right)}\left(r\right)\;r\;\mathrm{dr}+\overset{R}{\underset{\delta }{\int }}U_{z}^{\left(2\right)}\left(r\right)\;r\;\mathrm{dr}\right]=H_{c}\;\overset{\delta }{\underset{0}{\int }}U_{z}^{\left(1\right)}\left(r\right)\;r\;\mathrm{dr}$$

The difference between the three types of hematocrit diminishes, i.e. $H_{t}\approx H_{c}\approx H_{d}$ as δ/R→1. Tube and core hematocrits are forms that have been introduced exclusively in arteriolar blood flows.

Next, we rely on extensive experimental observations to quantify macroscopic hemodynamical properties [73,74] and develop the necessary correlations as follows. The Fähraeus-Lindqvist effect has been examined in detail by Pries and Secomb [73]. To this end, a series of experiments has been undertaken for different hemodynamical conditions (i.e., vessel diameter, discharged hematocrit, etc.). The non-Newtonian blood flow has been correlated with the well-known Hagen–Poiseuille law for laminar flow of Newtonian fluids in a tube. To match the blood flow data given in [73], the relationship of the flow-rate with the pressure-drop for different tube diameters is revised as follows

(19) $$Q\;=\;\frac{{\mathrm{JD}}^{4}}{128{\;\eta }_{\mathrm{rel}}{\;\eta }_{\mathrm{pl}}}$$

where Q and J are the volume flow rate and pressure gradient, respectively, and $\eta_{\mathrm{rel}}$ is the relative apparent viscosity ($=\eta_{\mathrm{app}}/\eta_{\mathrm{pl}}$). For a tube of a given diameter, Q, J and $\eta_{\mathrm{pl}}$ are measured, and Equation (19) is used to determine $\eta_{\mathrm{rel}}$. Then, we invoke the Fähraeus-Lindqvist phenomenon, i.e., the strong dependence of $\eta_{\mathrm{rel}}$ on the tube diameter and the discharged hematocrit ($H_{d}$), by the empirical law derived from the experiments conducted in glass tubes by Pries and Secomb [73]. This is a three-part relationship, which is relatively complicated, and hence, we have replaced it with our simplified form (the so called GDAT model) given by a single equation after a non-linear fitting on experimental data [73], which is provided by

(20) $$\eta_{\mathrm{rel}}\left(H_{d},D\right)\;=\;1.012\;{\left(\frac{3.2972}{D}\right)}^{\frac{20.29{\;H}_{d}}{D}}$$

where D is the tube diameter in μm. A more detailed description of this relationship and comparison with the original form of Pries and Secomb [73] (Equations (A11)–(A13)) is presented in Appendix B.

Equation (18) determines $H_{d}$. Measurements of the tube hematocrit are typically reported relative to the discharged hematocrit, i.e., the ratio $H_{t}/H_{d}$ is measured. This ratio is related to the Fähraeus effect and was measured experimentally by Pries et al. [74] for different values of the discharged hematocrit and a wide range of tube diameters. The relationship that describes this ratio is a function of $H_{d}$ and R (in μm) and is given by

(21) $$\frac{H_{t}\left(H_{d},R\right)}{H_{d}}={\;H}_{d}+\;\left(1-H_{d}\right)\left(1+1.7e^{-0.7R}-0.6e^{-0.022R}\right)$$

Equation (21) determines $H_{t}$ and combing its value with the value of $H_{d}$ and Equation (17) yields the CFL thickness. We discretize the above system of equations (Equations (1)–(21)) using central finite differences [75] in space and a backward Euler scheme for their time integration. Since we assume that the location of the interface (δ) is part of the solution, we deal with a moving boundary problem and hence we need to introduce a linear spatial transformation to solve the system in a fixed domain. The one-dimensional forms of the entire equation set, along with the boundary conditions, are presented in detail in Appendix A.

Although we present the steady-state values, our model is inherently transient, and thus all the predictions must be obtained through a transient solution. For all the examined cases, we assume an initially unperturbed and unyielded state ($\lambda_{\left|t=0\right.}=1$), while the stress and the velocity field are equal to zero ($\tau_{\mathrm{ve}\left|t=0\right.}=0,U_{z\left|t=0\right.}=0$), as it is described in detail in Appendix C.

## 3. Validation

Initially, we need to validate the predictions of our model against relevant flows reported in the literature. It must be pointed out that throughout the validation procedure, the examined flow conditions do not exactly match those of the corresponding experiments regarding the hematocrit value. As described in Section 2 and ref. [4], the parametrization of the TEVP model in this study corresponds to a rheological description of blood with a bulk hematocrit equal to 45%, and thus, the simulations are implemented by using a constant core hematocrit equal to this value. Most of the investigations assume a constant $H_{d}$ rather than a constant $H_{c}$. In the following Figures, blue solid curves represent the steady-state predictions of our TEVP model, while the symbols are data obtained by experimental studies.

**Figure 2:**
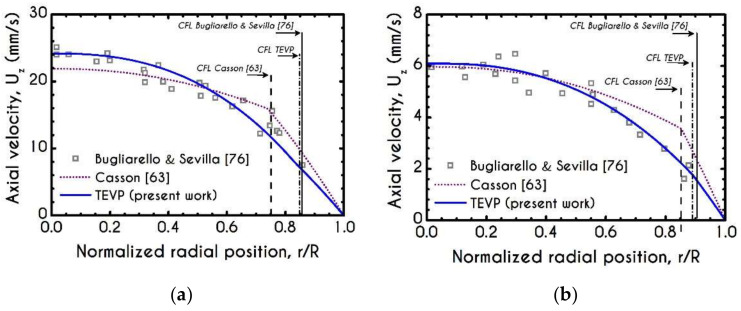

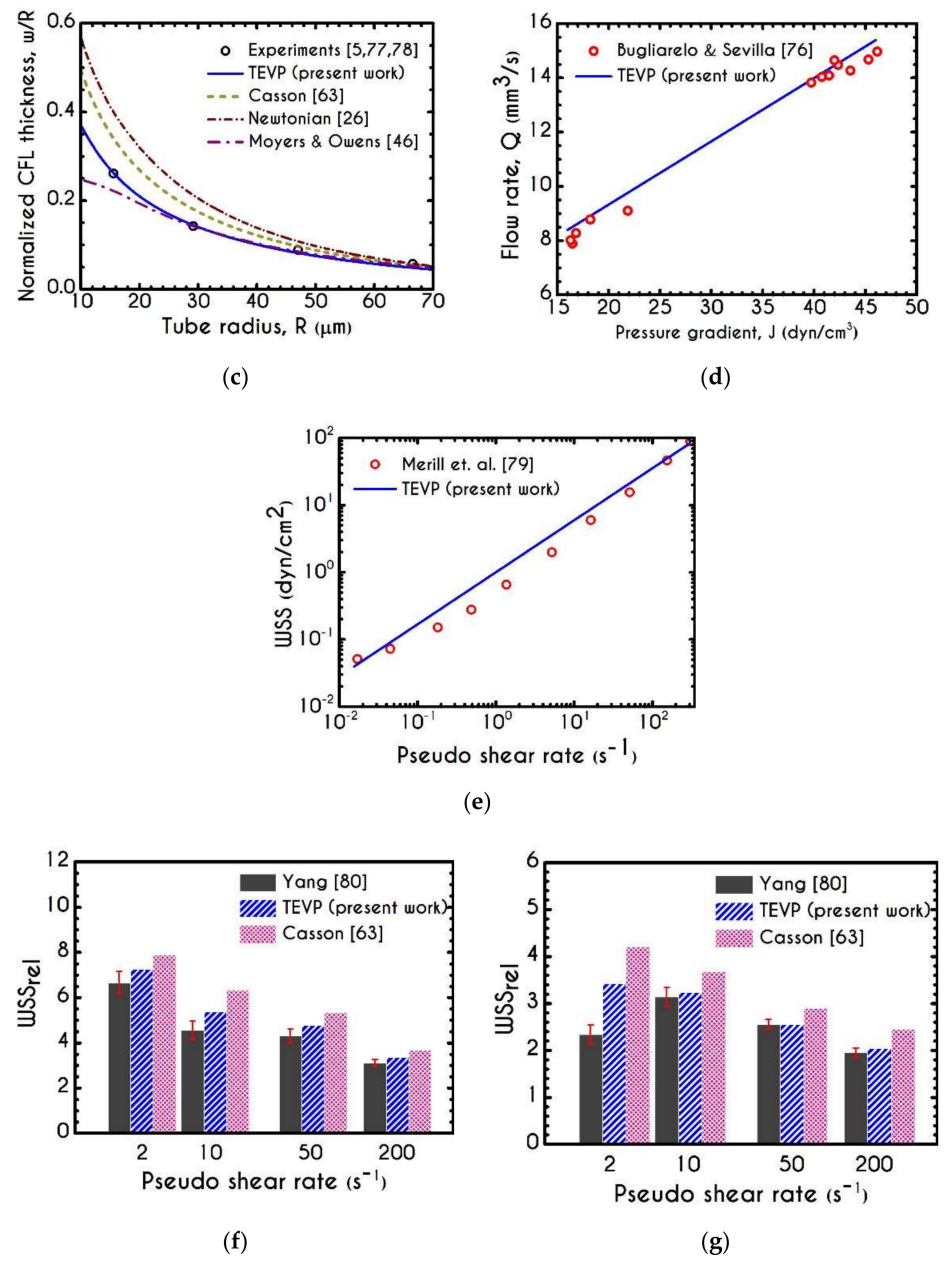
Validation of our model predictions with experimental data. Blue solid lines are steady-state simulation results of our model while open circles and cubes are experimental measurements for (**a**) velocity profile for R = 20 μm under the hemorheological conditions reported in Bugliarello and Sevilla [76], (**b**) velocity profile for R = 35 μm under the hemorheological conditions reported in Bugliarello and Sevilla [76], (**c**) Normalized thickness of CFL from various experimental investigations [5,77,78] accompanied with the predictions of a Newtonian model [26], the model of Casson [63] and Moyers-Gonzalez and Owens [46], (**d**) Total flow rate for the flow conditions reported in Bugliarello and Sevilla [76] for R = 20 μm, (**e**) Comparison of wall shear stress predictions with respect to pseudo shear rate $\bar{\dot{\gamma }}$ between TEVP and experimental observations from [79]. Comparison of relative wall shear stress ${\mathrm{WSS}}_{\mathrm{rel}}$ for various pseudo shear rates $\bar{\dot{\gamma }}$ between TEVP, Casson models and the experimental data from [80] for (**f**) R=15 μm and (**g**) R=50 μm.

Firstly, our predictions for velocity profiles are compared with experimental micro-PIV measurements of human blood flow in glass tubes. We select experimental data where flow conditions are close enough to ours regarding the core hematocrit $H_{c}$. Apparently, this is a challenging task since the literature lacks systematic works that present complete hemorheological data and hemodynamical measurements. To this end, we compare our steady-state results with those reported in the work of Bugliarello and Sevilla [76] for two cross-sections with different hemorheological conditions. Results under steady conditions are shown in Figure 2a,b for a discharged hematocrit equal to 40% in arterioles of 20 μm and 35 μm radius, respectively, while our simulation is under a constant core hematocrit value equal to 45%. Additionally, the experiments are conducted for an imposed mean axial velocity equal to $U_{\mathrm{mean}}=13\;\mathrm{mm}/s$ and $U_{\mathrm{mean}}=3.8\;\mathrm{mm}/s$ for 20 μm and 35 μm radius, respectively. Despite the somewhat different values of core hematocrit, our model is in excellent agreement with the experimental observations for both radii. We also provide the predictions of the Casson model [63] for the aforementioned experiments. We can observe that the predictions of the Casson model are not quantitatively good, especially for the case of R=35 μm, where the predicted velocity profile is more plug-like than that observed in the experiments. Additionally, the predictions of the inelastic Casson model are not close enough to the experimental observation regarding the CFL thickness. This is more obvious in Figure 2a,b where the CFL thickness is highlighted with light red, yellow and grey for the Casson, TEVP and experimental value, respectively. Indicatively, for the flow field in a microtube with a radius equal to 20 μm the predictions for the CFL thickness are 5.2 μm, 3.3 μm of the Casson and the TEVP models, while the corresponding values for the case of 35 μm radius, they are 5.25 μm and 3.85 μm, respectively. Our model is in excellent agreement with experimental observations of the CFL as Bugliarello and Sevilla [76] reported in their work that the CFL thickness is 3.3 μm and 3.36 μm for 20 μm and 35 μm tube radius, respectively. On the contrary, the predictions of the CFL thickness by the Casson model are not as good. In Table 4 we provide the predictions of our model for CFL thickness (w), flow rate (Q), Interfacial Normal (INS) and Shear Stresses (ISS), Wall Normal Stress (WNS) as well as the Wall Shear Stress (WSS) regarding the simulations with 20 μm and 35 μm radius, respectively.

In Figure 2c, we compare our numerical predictions for the dimensionless thickness of the cell-free peripheral layer with those experimentally observed [5,77,78] for a discharged hematocrit equal to 45%, and a pressure gradient $J=\;10^{4}\;\mathrm{Pa}/m$. The experimental points refer to tube diameters ranging from 30.8 μm and 132.3 μm with a pseudo-shear-rate varying between $5.38{\;s}^{-1}$ and $15.91s^{-1}$, respectively. Our model captures very accurately the observed decrease in the relative CFL thickness $\left(w/R\right)$, when the microtube radius increases, depicting an overall deviation of about 2%. This discrepancy is quite insignificant if one considers that our simulation encompasses a constant $H_{c}$, while the experiments employ a constant $H_{d}$. In the same Figure we provide the predictions of a Newtonian model [26], the model of Casson [63], and the model proposed by Moyers-Gonzalez and Owens [46] for the normalized CFL thickness. It is evident that our model provides more accurate results compared to those predicted by the other models. Although both the Newtonian and Casson models predict a similar behavior with that presented by our model, they both deviate considerably from the experimental values throughout, and the model of Moyers-Gonzalez and Owens [46] does not follow the trend of the experiments for radii below 30 μm. All these are absolutely normal since Newtonian and Casson models have only one and two adjustable parameters for the description of one or two out of five major mechanisms of the rheological response of blood. On the other hand the Owens’ model has eleven parameters as our TEVP model but it does not account for blood plasticity making it inaccurate at low shear-rates. Definitely, the coupled appearance of several viscous, elastic, plastic and thixotropic (RBC’s aggregation and disaggregation) phenomena and the underlying mechanisms necessitates the introduction of an adequate number of adjustable parameters to accurate represent their contribution in a thixotropic elasto-visco-plastic (TEVP) model and consequently the study of hemodynamics. 

Figure 2d illustrates the experimentally measured average volumetric flow rate of blood with respect to the imposed pressure gradient for 40% of discharged hematocrit. The experimental data are provided by the work of Bugliarello and Sevilla [76], and the only available data which were close enough to our rheological conditions were those for a microtube with a radius equal to 20 μm. The predicted flow-rate demonstrates a quite good agreement with the experimental data with a small overestimation at low pressure gradients. This deviation is mainly attributed to the presence of the migration effects, which may provoke a considerable deviation in the hematocrit of the experiment to that imposed by our simulation. In Figure 2e, we present the prediction of the Wall Shear Stress (WSS) as a function of the imposed pseudo shear-rate $\bar{\dot{\gamma }}\;\left(U_{\mathrm{aver}}/R\right)$ along with the experimental observations of the same quantity reported in [79]. We observe that our predictions demonstrate a similar behavior with that reported by Merill et al. [79] for a hematocrit equal to 40% and R = 100 μm. In any steady, axisymmetric flow, the shear stress is given by $\tau_{\mathrm{rz}}=\frac{\mathrm{Jr}}{2}$, where r is the radial position and J is the pressure gradient. Consequently, the shear stress on the wall demonstrates a linear dependence on both the pressure gradient and pseudo shear-rate. In experiments we observe a slight non-linearity which is attributed to either the failure of the experimental techniques since WSS it difficult to be measured accurately or the loss of axisymmetry (e.g., sedimentation of RBCs). Figure 2f,g show the predictions of the relative wall shear stress $\left({\mathrm{WSS}}_{\mathrm{rel}}\right)$ of the TEVP and Casson models [63] along with the experimental measurements of Yang [80] for horse blood flow with a hematocrit equal to 40%, in tubes with R equal to 15 μm and 50 μm, respectively. According to Yang [80], the ${\mathrm{WSS}}_{\mathrm{rel}}$ is defined as the wall shear stress obtained by the experiment $\left({\mathrm{WSS}}_{\exp}\right)$ divided by the wall shear stress exerted on the tube wall by the medium alone, i.e., blood in the absence of the RBCs $\left({\mathrm{WSS}}_{\mathrm{plasma}}\right)$. The latter quantity can be derived from the Poiseuille law as ${\mathrm{WSS}}_{\mathrm{plasma}}=2{\;\eta }_{\mathrm{pl}}U_{\max}/R$. In Figure 2f,g, the TEVP and the Casson parametrizations correspond to human blood with a hematocrit equal to 45% [49]. Considering the different values of hematocrit and the difference in the blood type, the predicted ${\mathrm{WSS}}_{\mathrm{rel}}$ of our TEVP model demonstrates a reasonably good agreement with the experimental data for both radii. The larger deviation between TEVP predictions and experimental data at low shear-rates can be attributed to the limitations of Equations (20) and (21). Both expressions are valid only in the high shear-rate limit. On the contrary, the Casson model always overestimates the TEVP prediction for the ${\mathrm{WSS}}_{\mathrm{rel}}$.

## 4. Numerical Results

This section presents the predictions of our one-dimensional steady-state simulations accounting for a continuous RBC-rich phase at the center of the tube and a plasma layer placed adjacent to the microtube wall. Arterioles are typically varying from 10 μm to 80 μm of radius, with a mean velocity of 2 mm/s. We perform a thorough parametric analysis under a wide range of microtube radii from 10 μm to 250 μm. The flow is driven by either a pressure gradient varying from $10^{2}\;\mathrm{Pa}/m$ to $10^{5}\;\mathrm{Pa}/m$ or a mean axial velocity $U_{\mathrm{mean}}$ ranging between 0.1 mm/s and 5 mm/s. The mean axial velocity is related to the total flow rate Q by $Q=U_{\mathrm{mean}}{\;\pi \;R}^{2}$. This model needs data from startup shear, cessation, and steady rheometry (or LAOS etc.), which can hardly be found in the open literature. Once the model parameters have been evaluated for a value of hematocrit (in this work is 45% given by McMillan’s experiments [55]), our hemodynamic simulations (e.g., flows in microcirculation) should consistently follow this restriction. Since blood flows only in the core region of a vessel or a glass tube, the core hematocrit (neither the tube nor the discharge hematocrit) should be equal to 45% in all cases. Typically, blood flow investigations in which microcirculatory phenomena are included impose a constant discharged hematocrit ($H_{d}$) rather than its core counterpart $\left(H_{c}\right)$.

### 4.1. Comparison with an Inelastic Model

**Figure 3:**
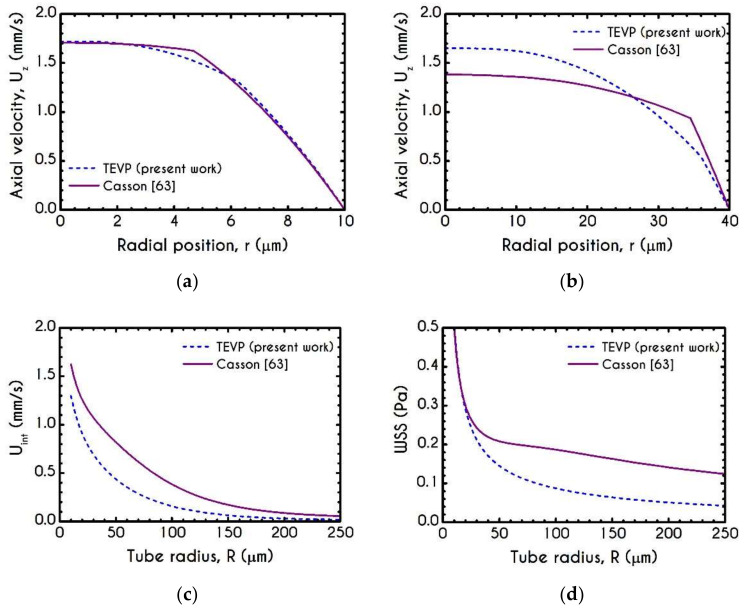
Comparison between the TEVP model with the inelastic model of Casson [63] regarding the velocity profiles within a microtube for (**a**) R =10 μm and (**b**) R =40 μm, (**c**) Interfacial axial velocity $U_{\mathrm{int}}$ as a function of the tube radius and (**d**) Wall Shear Stress (WSS) with respect to the radius of the microtube. Here, $H_{c}\;=45\%$ and $U_{\mathrm{mean}}\;=1\;\mathrm{mm}/s$.

It is very useful to compare the results of our model with that of the Casson inelastic model. In the following Figures, we present the predictions for both narrow and wide microtube with cross-section equal to 10 μm and 40 μm, respectively. The imposed pressure drop is $J=\;10^{4}\;\mathrm{Pa}/m$. We can observe that the predicted velocity profiles for TEVP and Casson models exhibit considerable discrepancies in the velocity profile (Figure 3a,b), interfacial axial velocities (Figure 3c), and wall shear stress (Figure 3d). Regarding the profile of the axial velocity, we can see that both models capture the non-parabolic profile of the blood flow—a behavior which is consistent with experimental observations in vitro [33]. In narrow microtubes, both TEVP and Casson models predict a blunted profile (Figure 3a), which gradually shifts towards parabolic as the radius of the tube increases (Figure 3b). The TEVP model predicts a smaller CFL thickness compared to that predicted by Casson, which in turn affects the maximum velocity prediction (Figure 3b). As we can see from Figure 3c,d, the interfacial velocity $U_{\mathrm{int}}$ and the WSS predicted respectively by the TEVP model are lower than those predicted by the inelastic model for R= 40 μm.

### 4.2. Effect of Proteinic Elasticity in the Plasma Layer

**Figure 4:**
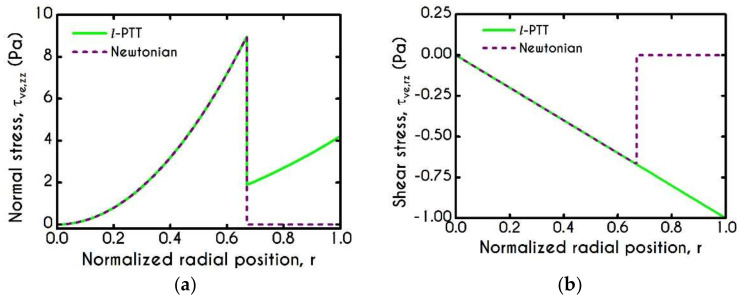
(**a**) Normal viscoelastic stress $\tau_{\mathrm{ve},\mathrm{zz}}$, and (**b**) Shear viscoelastic stress $\tau_{\mathrm{ve},\mathrm{rz}}$ for R = 20 μm, $J\;=\;5\times 10^{5}\;\mathrm{Pa}/m$, and $H_{c}=0.45$.

Since the non-elastic model of plasma is used extensively in blood flow modeling, we compare its predictions against those of our l- PTT model to estimate the error involved in neglecting the elastic contribution of plasma proteins. Although plasma exhibits much smaller elasticity than blood, as indicated by the shorter relaxation time of plasma $\lambda_{\mathrm{pl}}$ (see Table 1 and Table 3), it can induce significant differences in the stress field response. The latter is mainly affected by high shear rates or extensional phenomena. These phenomena are absent in one-dimensional flows but are substantial in more complex flows such as hemodynamics in arteriole branching, saccular aneurysms, and arterial bifurcations [42,81]. The scope of this work is limited to examining the crucial features of blood in one-dimensional flows offering a consistent model for more complex flows and not to probe extensional flows. Figure 4 illustrates the normal and shear viscoelastic stress profiles for abnormal hemodynamical conditions characterized by high pressure-gradients J. The prediction of normal and shear stress in the plasma phase is much higher than that of a Newtonian model, as depicted in Figure 4a.

### 4.3. Effect of Radius

Cell migration is an essential part of the mechanism behind both the development of the CFL formation and the Fåhraeus effect, as it has been stated in the previous sections. These phenomena are mainly governed by excluded volume effects [82,83] and cell-cell interactions or collisions [84,85], which are enhanced by aggregation [86,87]. Erythrocytes appear in two structural forms of individual cells and aggregated cells, the distribution of which is strongly related to the stress field applied. The dynamic equilibrium shifts toward more individual cells when the applied shear-rate increases, which affects the overall configuration of the velocity field, inducing a distinguishable difference between narrow and wide tubes.

**Figure 5:**
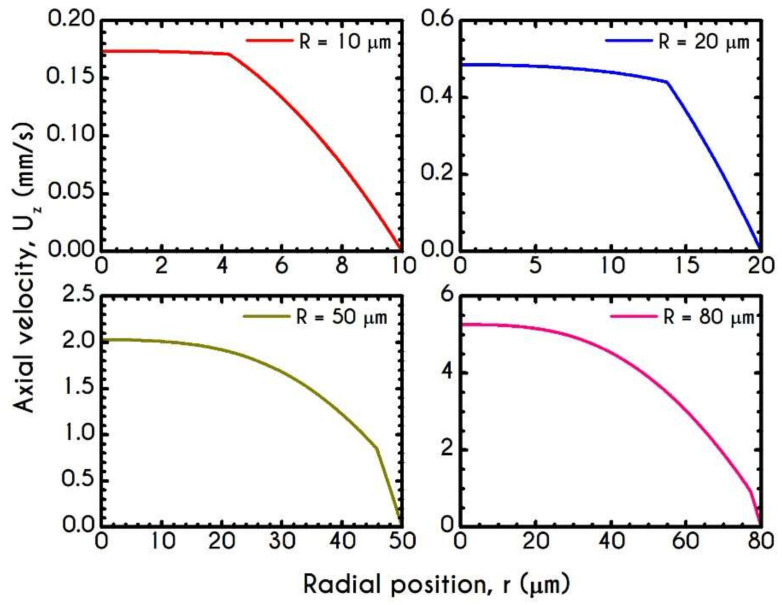
Axial velocity profile, $U_{z}\left(r\right)$, along the radial position for the tube radius equal to 10 μm, 20 μm, 50 μm, and 80 μm, for $J\;=\;10^{4}\;\mathrm{Pa}/m$ and $H_{c}=0.45$.

Figure 5 presents the profile of the axial velocity $U_{z}$ along the radial position r of the tube, for radius ranging from 10–80 μm for an applied pressure gradient equal to $J=10^{4}\;\mathrm{Pa}/m$. Under these conditions, the dynamics of the system in narrow microtubes reveals that the velocity is more plug-like compared to wider ones, which is enhanced by the fact that in small tubes, aggregation is promoted [86]. The discontinuity in the shear rate between the fluid in the core region and that in the peripheral layer indicates the presence of the CFL. As the radius increases, the applied shear rates increase too and lead to a progressive rouleaux breakdown into individual cells promoting a more parabolic profile, as illustrated in Figure 5. In large tubes, the migration effects are less intense, and hence the CFL thickness is significantly smaller than that predicted for the narrower microtubes. When the applied shear stress is higher than the yield stress, the rouleaux network is broken, and the blood is free to flow like a liquid. This can also be evaluated by the structure parameter λ, which constitutes an indicator for the instantaneous state of blood within the tube.

**Figure 6:**
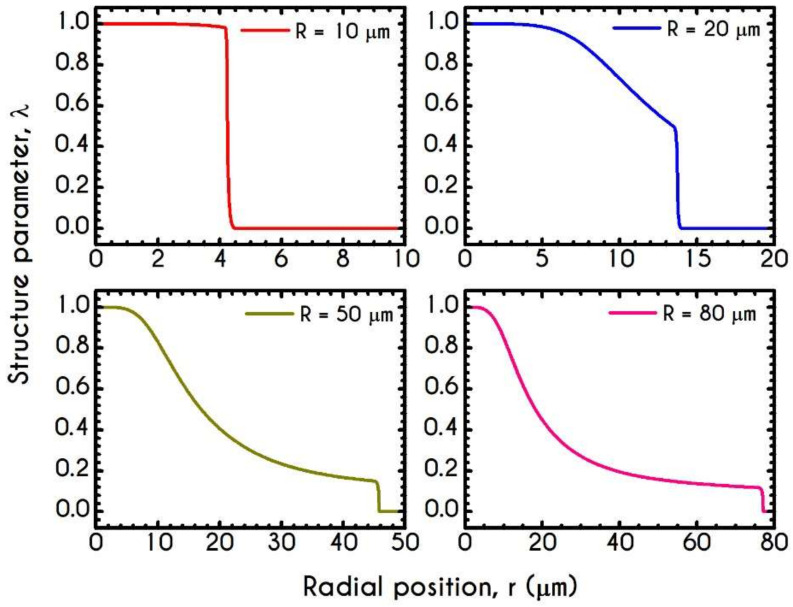
Distribution of the structure parameter λ along the radial position for tube radius equal to 10 μm, 20 μm, 50 μm, and 80 μm, for $J\;=\;10^{4}\;\mathrm{Pa}/m$ and $H_{c}=0.45$.

Figure 6 demonstrates this thixotropic variable justifying the previous assertion. In general, blood is predicted to be in a fully structured state near the center of the tube, the extent of which is highly affected by the applied shear-rates. For very narrow tubes, such as those of R =10 μm where the cross-section is comparable to RBC diameter, we observe two distinct zones with a sharp transition between them. The first zone is the RBC-rich region characterized by a fully structured state, and the second one is the plasma phase with λ = 0. It is evident that in this case, aggregation in the core is quite intense as blood does not demonstrate any change from its initial state and remains fully structured at steady-state conditions. The higher the imposed pseudo-shear-rate, the narrower is the plug flow region where blood depicts a fully structured form. At the center of the tube, λ is always equal to unity since the shear-rate is zero there. On the contrary, as the distance from the center increases, the shear-rate attains higher values. Consequently, the breakdown term dominates, enforcing an abrupt decrease in blood aggregates. This behavior is apparent in microtubes with wider cross-sections where the aggregation of RBCs is relatively weak. In comparison with the same blood flowing in smaller microtubes, λ attains lower values.

**Figure 7:**
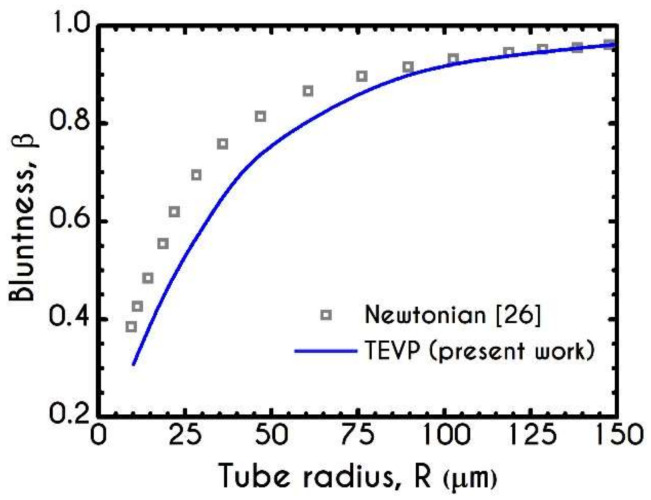
Variation of the bluntness parameter β with the tube radius, for $J\;=\;10^{4}\;\mathrm{Pa}/m$.

An indicator that demonstrates the deviation of the blunted velocity profile from a parabolic one is the parameter β. It characterizes the bluntness of the velocity profile in the core by correlating the average viscosities in the two phases given by

(22) $$\beta =\;\frac{\bar{\eta_{b}}/\bar{\eta_{p}}}{1-w^{2}\left(1-\bar{\eta_{b}}/\bar{\eta_{p}}\right)}\;$$

where $\bar{\eta_{b}}$ and $\bar{\eta_{p}}$ are the mean viscosity of the core region and the plasma phase, respectively. The mean shear viscosity of blood, $\bar{\eta_{b}}$, is determined as $\int_{o}^{\delta }\left(\frac{\tau_{\mathrm{ve},\mathrm{rz}}}{{\dot{\gamma }}_{\mathrm{rz}}}\right)\mathrm{rdr}/\int_{0}^{\delta }\mathrm{rdr}$. Particularly, a value of β close to zero indicates that the velocity profile is nearly plug, while the pure parabolic profile is indicated by a β equal to unity, i.e., viscosities in the core and the plasma layer are the same and equal to the bulk viscosity. Figure 7 reveals that the bluntness parameter β increases as the diameter of the tube increases; in other words, the deviation from the parabolic profile decreases as the tube diameter increases. In the same Figure, we also illustrate the predictions of a two-phase model with a Newtonian representation for both blood and plasma. As it is expected, our predictions depict a considerable deviation from the pure Newtonian modeling, demonstrating a blunter profile for values of radius below 100 μm. The two curves converge above 100 μm of radius, predicting an almost equal bluntness parameter as the velocity profile reaches the parabolic form. Notably, our model predicts that the bluntness of the velocity profile is increased when β drops from 0.9 to 0.3 as well as the tube radius reduces from 120 μm to 10 μm. Further, for 100 μm to 150 μm in radius, the parameter β is increased from 0.9 to 0.96. For further increase of the radius, β asymptotically reaches unity. Thus, the velocity profile becomes more parabolic when the tube diameter is increased.

**Figure 8:**
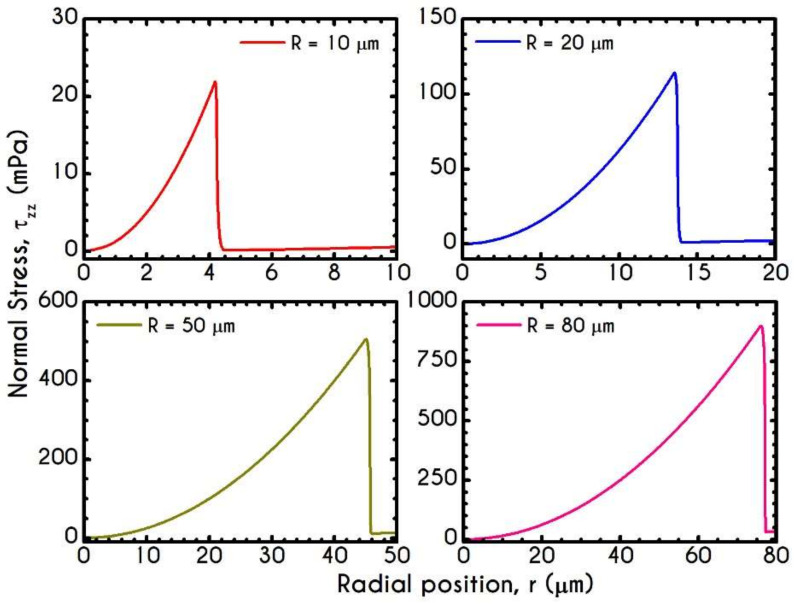
Normal viscoelastic stress $\tau_{\mathrm{ve},\mathrm{zz}}$ along the radial position r for the tube radius R equal to 10 μm,  20 μm, 50 μm, and 80 μm for $J\;=\;10^{4}\;\mathrm{Pa}/m$ and $H_{c}=0.45$.

**Figure 9:**
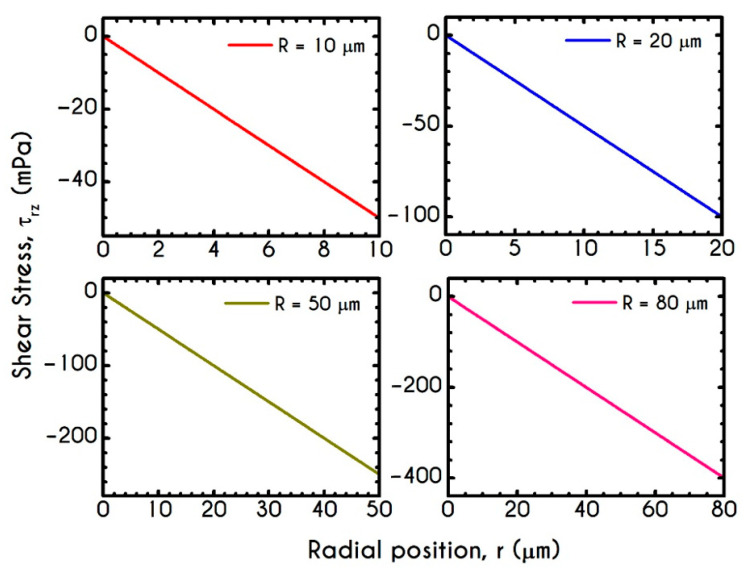
Shear viscoelastic stress $\tau_{\mathrm{ve},\mathrm{rz}}$ along the radial position r for tube radius R equal to 10 μm,  20 μm, 50 μm, and 80 μm for $J\;=\;10^{4}\;\mathrm{Pa}/m$ and $H_{c}=0.45$.

Figure 8 and Figure 9 demonstrate the prediction of the viscoelastic stresses for radius equal to 10 μm, 20 μm, 50 μm, and 80 μm with an imposed pressure gradient of $J\;=\;10^{4}\;\mathrm{Pa}/m$. The normal component of the stress tensor (Figure 8) depicts the same pattern for all examined cases. As the shear rate gradually increases along the radial position, $\tau_{\mathrm{ve},\mathrm{zz}}$ progressively increases from zero to a maximum value, which is strongly dependent on the local shear rates. The pick of normal stress component is observed at the blood/plasma interface, followed by an abrupt decrease within the plasma phase. Within the CF Layer, the normal stress is finite but relatively insignificant compared to that in the core region for the imposed pressure gradient. A considerable contribution of normal stress in the plasma phase should occur under extremely high shear rates. However, the viscoelastic behavior must not be underestimated. The only observable differentiation in normal stress predictions for different R is the maximum value at the blood/plasma interface as well as the phase change location, i.e., the CFL thickness w. The maximum value for each case at the blood/plasma interface is 21 mPa, 112 mPa, 525 mPa, 914 mPa for 10, 20, 50, and 80 μm in radius, respectively. In Figure 9, we present the spatial variation of the shear component of the viscoelastic stress tensor $\tau_{\mathrm{ve},\mathrm{rz}}$ for the same rheological conditions. The latter refers to the total shear stress applied to the system, as we assumed a negligible contribution from the solvent, highlighting a linear distribution along the radius of the microtube. Similarly to the normal stress prediction, the magnitude of $\tau_{\mathrm{ve},\mathrm{rz}}$ increases with r and R as a consequence of the appearance of higher values of shear rates. By comparing the maxima of normal and shear components, we observe that $\tau_{\mathrm{ve},\mathrm{zz}}$ is lower than $\tau_{\mathrm{ve},\mathrm{rz}}$ up to a tube radius equal to 20 μm. On the contrary, for cross-sections higher than 50 μm the normal stress contributes significantly to the total stress and overcomes the contribution of the shear component. Most of the blood constitutive modelling investigations do not present the normal stress prediction, and hence we are not able to make a comparison with other studies. Varchanis et al. [56] in their work reported a significant contribution of normal stress in simple shear tests and compared their findings with those predicted by the ML-IKH model [61], which was found to have similar behavior. The presence of normal stresses in suspensions is attributed to the intense interaction between the particles, whereas in plasma to protein stretching. Similar arguments are presented by Mall-Gleissle et al. [88] for suspensions with viscoelastic matrix fluids.

**Figure 10:**
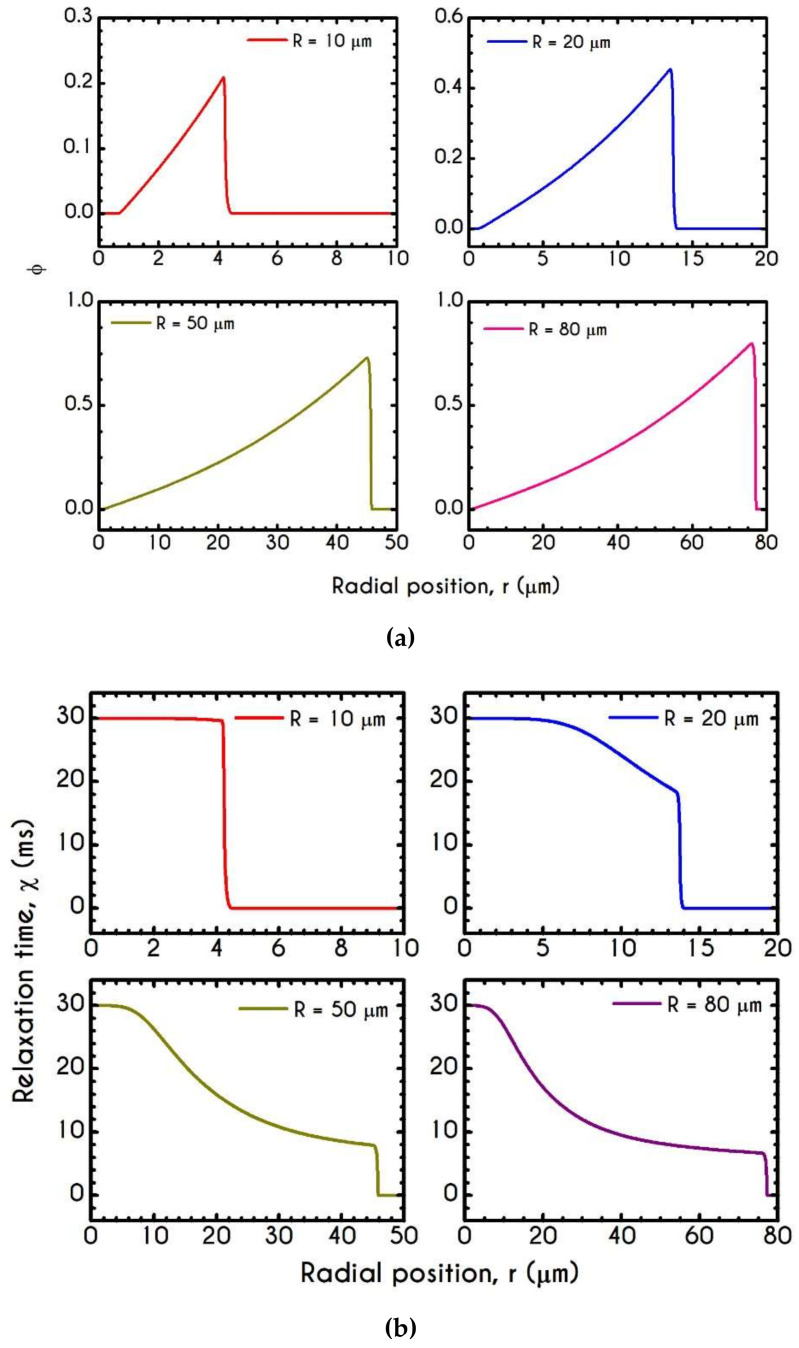
Distribution of (**a**) Parameter φ and (**b**) Relaxation time χ along the radial position r for tube radius of 10 μm,  20 μm, 50 μm, and 80 μm for $J\;=\;10^{4}\;\mathrm{Pa}/m$ and $H_{c}=0.45$.

The condition of whether blood is yielded or unyielded is defined through the von Mises criterion via the parameter φ. Figure 10a presents the spatial variation of φ along the radial position r for microtubes of different radii. If the stress components present in the system are large enough to satisfy the von Mises yielding criterion, the quantity φ acquires non-zero values indicating that the yield stress has been exceeded and blood is fluidized. On the contrary, a zero value of φ indicates unyielded blood, like this depicted in the case with R = 10 μm. Near the center of this tube, the stresses are insignificant, resulting in an unyielded region, the size of which depends on the radius of the tube for the same imposed pressure gradient. Clearly, φ=0 in the CFL. Figure 10b presents the relaxation time $\chi =\frac{\eta_{t}\left(\lambda \right)}{G}$ variation along the radial position r. It is obvious that it follows similar dynamics with that depicted by the structure parameter due to our assumption that plastic viscosity depends on the instantaneous state of blood, and thus it is potentially a thixotropic property.

**Figure 11:**
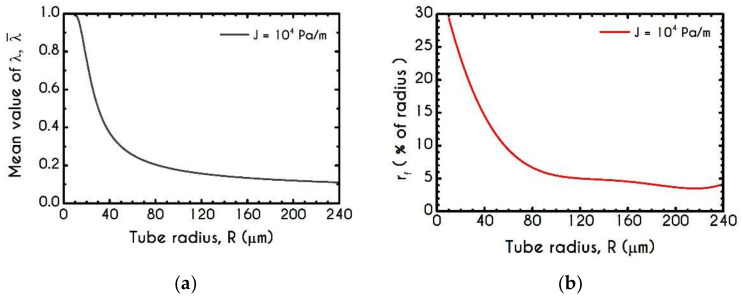

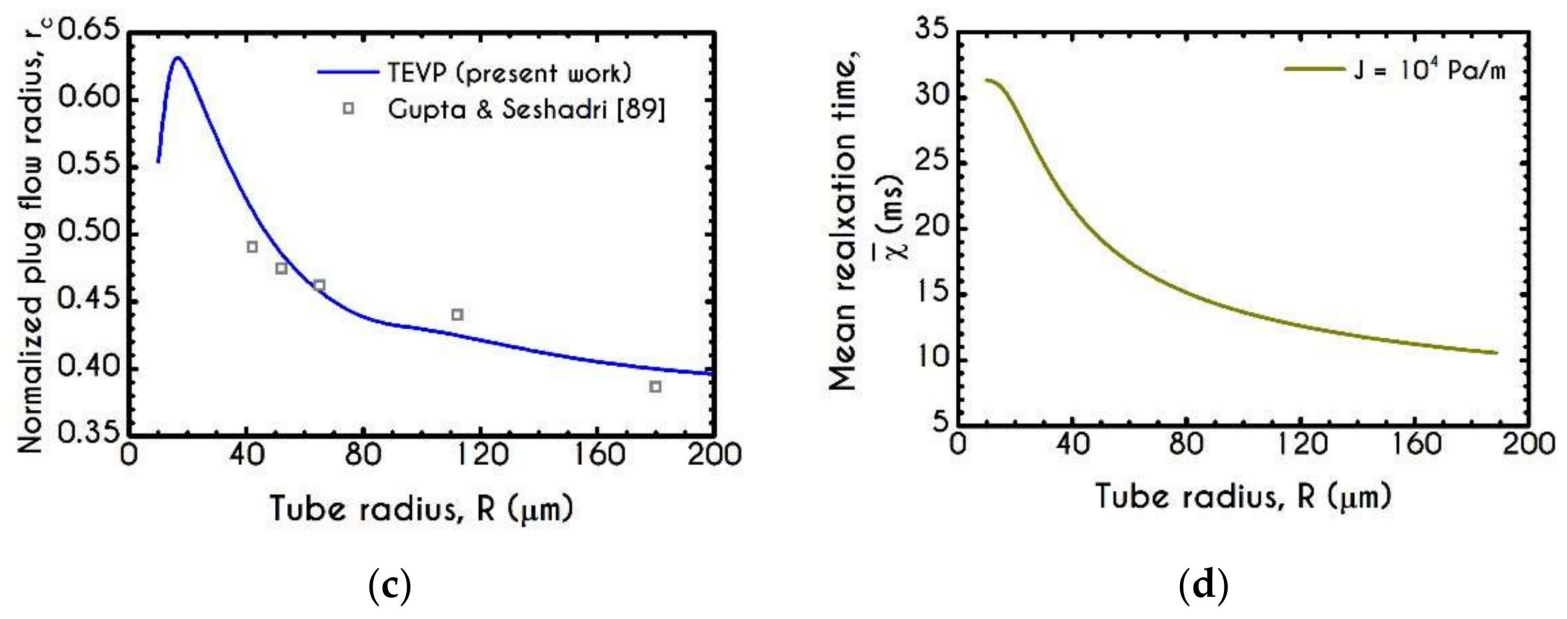
(**a**) Mean structure parameter $\bar{\lambda }$, (**b**) Fully structured region $r_{f}$ as a percentage of the radius of the tube R, (**c**) Normalized plug velocity size $r_{c}$ along with the experimental data of Gupta and Seshadri [89] for the same quantity and (**d**) Mean relaxation time $\bar{\chi }$ as a function of the microtube radius. In all cases $J\;=\;10^{4}\;\mathrm{Pa}/m$ and $H_{c}=0.45$.

As we have already mentioned, the instantaneous state of the blood is defined through the parameter λ. In the current formulation, we have assumed that our model includes a stress-controlled structural parameter in the sense that the thixotropic behavior of blood is controlled by the applied stresses via the von Mises criterion. Figure 11a depicts the steady-state values of the mean structural parameter $\bar{\lambda }$ in the core region of the tube, as a function of its radius. We observe a continuous deconstruction of the RBCs aggregates as the radius of the tube increases due to higher shear and extensional stresses (see Figure 8 and Figure 9). Across the whole range of the examined radii, λ experiences a reduction from λ = 1 for R = 10 μm to λ = 0.1 for R = 240 μm, with a higher decrease up to 100 μm, while beyond this point, the average structural parameter continuously seems to approach an asymptote. Figure 11b shows the variation of the fully structured fraction of the core region, where λ is equal to unity. In particular, we use the quantity $r_{f}$, which stands for the percentage of the structured region out of the radius of the microtube. Also, it is used for quantifying the extension of the RBC aggregation. Blood aggregability exhibits an almost continuous reduction as blood flows in larger tubes. Indicatively, in a microtube of radius equal to R = 10 μm, we observe that fully structured material extends to 30% of R. $r_{f}$ follows an abrupt reduction when R= 80 μm for which its corresponding value is 6%. As the radius increases further to 240 μm, $r_{f}$ asymptotes to 5% approximately.

A comparison of our model results against plug flow predictions can be made by invoking the results of Gupta et al. [89]. They reported the experimental observations of the velocity profiles in microtubes. In particular, they measured the region for which the velocity profile follows a plug pattern. Figure 11c reports the experimental data of Gupta et al. [89] along with our predictions for the normalized plug flow radius $r_{c}$ as a function of the radius of the microtube. We observe an excellent agreement, from 40 μm up to 180 μm of radius with an overall deviation of about 4.5%. For tubes with a radius less than 40 μm, a non-monotonic behavior can be observed. This is mainly attributed to the variation of CFL with the radius of the tube, the width of which varies in a similar manner as $r_{c}$. At this point, it is necessary to underline the difference of $r_{c}$ and $r_{f}$. The former is a feature of the velocity profile and highly correlated with the thickness of the CF Layer, while the latter represents the region where the RBCs are structured in aggregated forms.

In Figure 11d, we observe the prediction of the steady blood mean relaxation time $\bar{\chi }=\bar{\eta_{t}}/G$ as a function of the microtube radius R. It exhibits a continuous reduction as the radius of the tube increases. From R=10 μm to R=100 μm the relaxation time experiences a steep decrease from $\bar{\chi }=31.4\;\mathrm{ms}$ to $\bar{\chi }=13.7\;\mathrm{ms}$, while for wider radii, the mean value approaches an asymptote. This behavior comes mainly from the fact that we have considered a plastic viscosity that depends on the instantaneous state of blood. Thus, as the radius increases, the microstructure of blood is more disintegrated.

**Figure 12:**
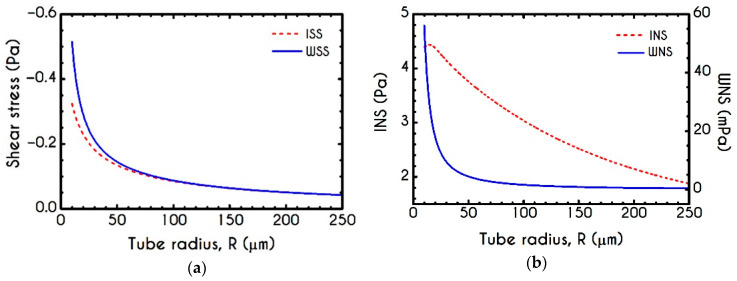
(**a**) Interfacial (ISS) and Wall (WSS) Shear Stresses and (**b**) Interfacial (INS ) and Wall (WNS) Normal Stresses as a function of the tube radius R for $U_{\mathrm{mean}}\;=\;1\;\mathrm{mm}/s$ and $H_{c}=0.45$.

One of the most important quantities in blood flow studies is the Wall Shear Stress $\left(\mathrm{WSS}\right)$, which is the total shear stress exerted on the microtube’s wall. A proper calculation of WSS has an exceptional role, especially when blood flows in vivo because it is the stress applied on the internal Endothelial Cells (EC) surface. Vascular operations such as biochemical reactions are considerably affected by the WSS as it has been proved to be directly associated with Nitric Oxide (NO) production [43] and calcium activation in Smooth Muscle Cells (SMC) [90] by triggering the biochemical reactions that take place in vascular beds, leading to the regulation of vascular tone [91]. Although in one-dimensional blood flows, the prediction of WSS is not complex, we offer a consistent model for a proper prediction of WSS in more complicated flows. Figure 12 shows the distribution of ISS, WSS, INS, and WNS for different cross-sections of the arteriole for an imposed mean axial velocity equal to $U_{\mathrm{mean}}=1\;\mathrm{mm}/s$. As it is expected, the wall shear stress demonstrates a non-linear dependence on arteriole radius. As we have neglected the solvent contribution in the plasma phase, the predicted WSS is related to the pure viscoelastic contribution of the proteinic phase. For a fixed mean velocity within the tube, the ISS and WSS demonstrate a gradual decrease until an asymptotic behavior is achieved. Our model predicts a significant contribution of normal stress for both interfacial and wall locations, which would be crucial for more complex blood flows such as arterial bifurcation or saccular aneurysms where the extensional phenomena are intense enough to promote the development of $\tau_{\mathrm{ve},\mathrm{zz}}$. Both shear and normal stress components can be expressed as a function of the tube radius R in μm through a non-linear relationship of the form

(23) $$s_{n}\left(R\right)\;={\;a}_{n}\;+b_{n}R+{\;c}_{n}R^{2}$$

with n=1,2,3,4 refer to ISS,WSS,INS, and WNS respectively. The corresponding coefficients are presented in Table 5.

**Figure 13:**
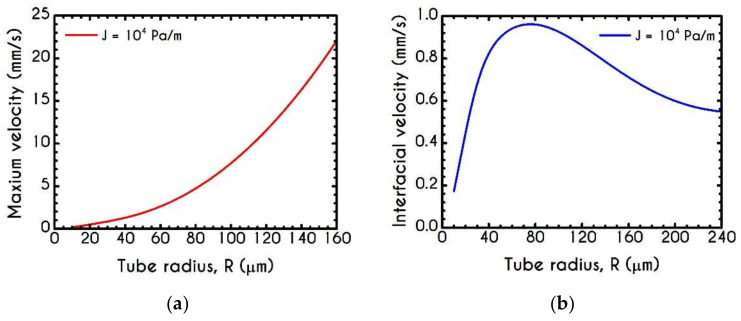
(**a**) Maximum axial velocity $U_{\max}$, and (**b**) Interfacial axial velocity $U_{\mathrm{int}}$ as a function of tube radius R for $J\;=\;10^{4}\;\mathrm{Pa}/m$ and $H_{c}=0.45$.

Figure 13 presents the maximum velocity $U_{\max}$ and interfacial velocity $U_{\mathrm{int}}$ as a function of the microtube radius. As blood flows under a constant pressure gradient in larger tubes, the axial velocity attains higher values, and hence the predicted maximum value demonstrates a continuous increase (Figure 13a). However, interfacial velocity does not exhibit a monotonic behavior, as illustrated in Figure 13b. This is attributed to the reduction of the aggregation effects, which enhance the transition of the velocity profiles from plug to parabolic ones. This behavior can also be justified by the predictions of velocity profiles for the two-phase blood flow in narrow tubes [27,46].

**Figure 14:**
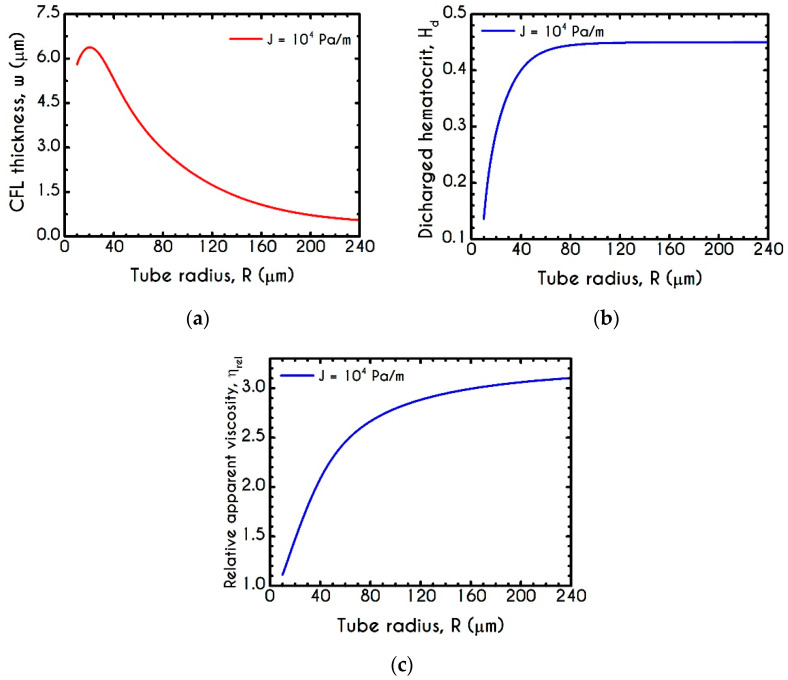
(**a**) Cell-free layer thickness w, (**b**) discharged hematocrit $H_{d}$, and (**c**) the relative apparent viscosity $\eta_{\mathrm{rel}}$, as a function of tube radius R for $J\;=\;10^{4}\;\mathrm{Pa}/m$ and $H_{c}=0.45$.

Migration effects in microcirculation are more clearly visible through the variation of the CFL thickness prediction and the evaluation of the discharged hematocrit. Figure 14 demonstrates the steady-state values of w, $H_{d}$ and $\eta_{\mathrm{rel}}$ as a function of the tube radius R for $J\;=\;10^{4}\;\mathrm{Pa}/m$ and $H_{c}=0.45$. Apparently, the tendency of the erythrocytes to migrate towards the center of the tube implies an interrelation between the hemodynamical properties such as the CFL thickness, the discharged hematocrit, and the apparent viscosity. As the tube radius decreases from 250 μm down to 20 μm, the apparent viscosity ($\eta_{\mathrm{rel}}$) drops due to the CFL formation next to the wall, leading to a decrease in discharged hematocrit. As the location of the CFL interface is coincident with the region of the highest shear rate within the flow, the presence of such a layer can significantly reduce the apparent viscosity. Irrespectively of the pressure drop used in the current simulation, both quantities are affected considerably by the radius R. Figure 14a demonstrates a non-monotonic behavior of the computed cell-free layer width across the wide range of the examined cross-sections. CFL is determined by a balance between lateral migration and mass diffusion caused by cell–cell interactions. To this end, as the tube diameter decreases below a specific value, red cell migration becomes restricted due to strong interaction between RBCs in the core. This is the reason why we observe the non-monotonic behavior for the microtubes of radius below 20 μm. Although our continuum model agrees very well with the empirical observations of Pries et al. [92], it may cease to be valid once the tube radius becomes comparable to the diameter of the individual RBCs. To validate our prediction, we invoke the results of a related study in which a different constitutive model for the description of blood rheology has been used. In that work, Moyers-Gonzalez and Owens [46] conducted blood flow simulations regarding the CFL thickness under various hemodynamical conditions. Unfortunately, we are not able to completely compare our model results with those reported in their work because they simulated blood flows with different values of the discharged hematocrit and not with its core counterpart. As we impose a constant value of the core hematocrit in the current work, the only comparable CFL thickness is that predicted for a microtube with a radius of about 27 μm. At this radius, the discharged hematocrit predicted by our model is almost equal to 0.2 which is one of the examined values of the discharged hematocrits in [46]. Our model predicts w=5.74 μm, while Moyers-Gonzalez and Owens reported a value equal to 5.68 μm, i.e., a discrepancy of only 1.04%. As the radius increases from 20 μm to 250 μm the CFL thickness follows a monotonic decrease as it is expected. When a radius lies in this range, migration phenomena become inappreciable leading to a more parabolic velocity profile and hence the apparent viscosity resembles that given by the Poiseuille law. These phenomena seem to be quite weak above a radius of 180 μm where an asymptote is approached. For a more detailed description of the interplay between the apparent viscosity and the CFL formation, the reader is referred to Appendix A. Determining the CFL thickness is of significant importance not only for a two phase blood flow simulation but also for other processes. To this end we provide a mathematical expression for the evaluation of w in μm as a function of the microtube radius R given by

(24) $$w\left(R\right)=\;\frac{w_{1}+w_{2}R}{1+w_{3}R+w_{4}R^{2}}\;$$

where $w_{k}$ with k=1,4 are adjustable parameters, which are presented in Table 6. The radius R is in μm.

Figure 14b describes the effective migration through the display of the $H_{d}$ as a function of the radius of the microtube, corresponding to a fixed value of $H_{c}$. It is obvious that a constant $H_{c}$ does not imply a constnant $H_{d}$ for small radii. As it is expected, the increased CFL thickness in a narrower microtube indicates intensification of the migration of the erythrocytes, yielding a considerably lower value of the discharged hematocrit corresponding to a core hematocrit equal to 45%. Indicatively, from 10 μm up to 67 μm of radius, we observe a remarkable increase of $H_{d}$ from an extremely low value of about 0.135 to 0.44, respectively. Consequently, it is safe to claim that above a 70 μm radius, the migration effects are relatively insignificant when blood flows in vitro.

### 4.4. Effect of Pressure Gradient

Another significant model parameter is the flow rate or the applied pressure-gradient because it impacts the velocity profile [93] and, consequently, the instantaneous state of blood microstructure. In the following Figures, we demonstrate the effect of the imposed pressure gradient J on the velocity profile, the viscoelastic stress distribution, and the steady structure parameter λ. The simulation refers to a microtube with a radius of R = 20 μm while the pressure gradient ranges from $10^{2}\;\mathrm{Pa}/m$ to $10^{5}\;\mathrm{Pa}/m$ with a constant core hematocrit equal to $H_{c}=0.45$.

**Figure 15:**
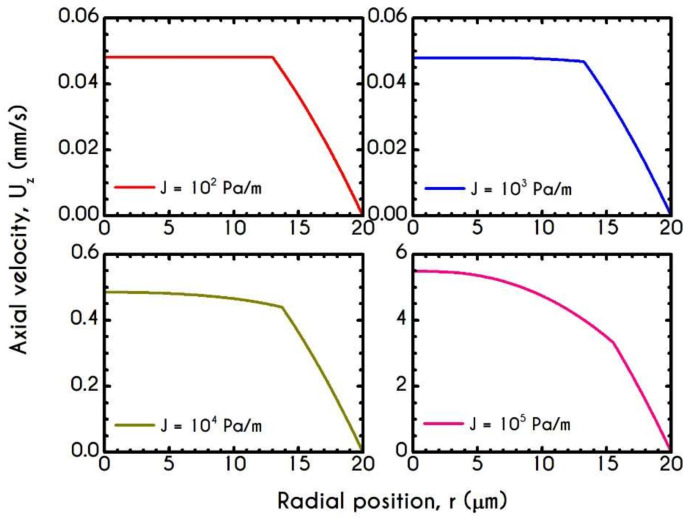
Axial velocity profile along the radial position for pressure gradient J equal to $10^{2}\;\mathrm{Pa}/m$, $10^{3}\;\mathrm{Pa}/m$, $10^{4}\;\mathrm{Pa}/m$, and $10^{5}\;\mathrm{Pa}/m$ for R = 20 μm and $H_{c}=0.45$.

Figure 15 demonstrates the steady-state profile of the axial velocity $U_{z}$ along the radial position r, when blood flows under the aforementioned rheological conditions. As it is expected, the imposed pressure gradient affects the bluntness of the velocity profile, indicating a plug-like flow for and a more parabolic-like behavior for $J=\;10^{5}\;\mathrm{Pa}/m$. However, the variation is limited to profile skewness and the magnitude of the maximum velocity. Particularly, as the bluntness of the profile increases, the axial velocity field acquires higher values. However, the CFL thickness remains nearly constant, as it is implied by the experimental observations of Pries et al. [73], who argued that migration effects are not significantly affected by the applied shear rates.

**Figure 16:**
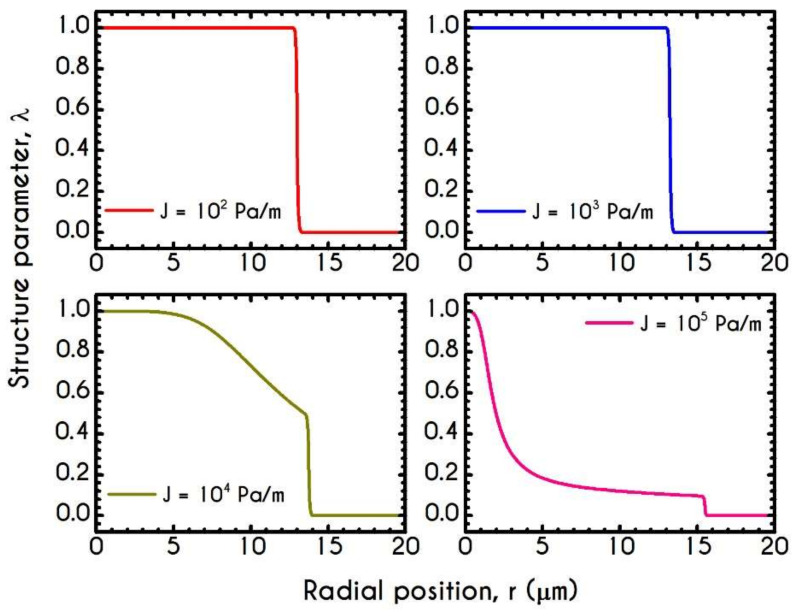
Distribution of the structure parameter λ along the radial position for different pressure gradients J equal to $10^{2}\;\mathrm{Pa}/m$, $10^{3}\;\mathrm{Pa}/m$, $10^{4}\;\mathrm{Pa}/m$, and $10^{5}\;\mathrm{Pa}/m$, for R = 20 μm and $H_{c}=0.45$.

To further elucidate the impact of the pressure gradient on the rheological behavior of blood, we present the steady-state values of the structure parameter distribution along the radial position of the tube r (Figure 16). For all imposed pressure gradients, the blood is initially at rest with $\lambda_{\left|t=0\right.}\;=\;1$. Beginning from the same state, we distinguish four different responses of the blood regarding its final microstructural configuration. When $J=\;10^{2}\;\mathrm{Pa}/m$ and $J=\;10^{3}\;\mathrm{Pa}/m$, blood does not exhibit any change from its initial state, meaning that the stress has not exceeded the yield-stress value to disintegrate the rouleaux. Thus, in these cases, we have two distinct areas, a core region with λ=1, and a plasma phase with λ=0. Further increase of pressure gradient causes a partial deconstruction of rouleaux. Particularly, for a pressure gradient equal to $J=\;10^{4}\;\mathrm{Pa}/m$, λ demonstrates a monotonic decrease from 1 to 0.48. It is obvious that the total stress near the center of the tube does not surpass the blood yield stress, and thus, blood remains in an unstructured state. Interestingly, with an imposition of a pressure gradient of one order of magnitude higher than the previous one, the thixotropic parameter λ experiences a steep decrease caused by the higher stress applied. In the vicinity of the blood/plasma interface, where the shear rates are high, the structure parameter approaches an asymptote at a low value. The size of the region where λ has an almost constant value is associated with the imposed pressure-gradient. The higher the J, the wider the region where λ is maintained at a constant low value. This is more obvious in the case with $J=\;10^{5}\;\mathrm{Pa}/m$, where the microstructure of blood demonstrates a significant collapse. Here, blood has a constant value of about λ= 0.13 from 8 μm to 13.6 μm. In any case, blood never becomes fully unstructured in the core region, i.e., λ never reaches 0, irrespective of the intensity of the imposed pressure gradients.

**Figure 17:**
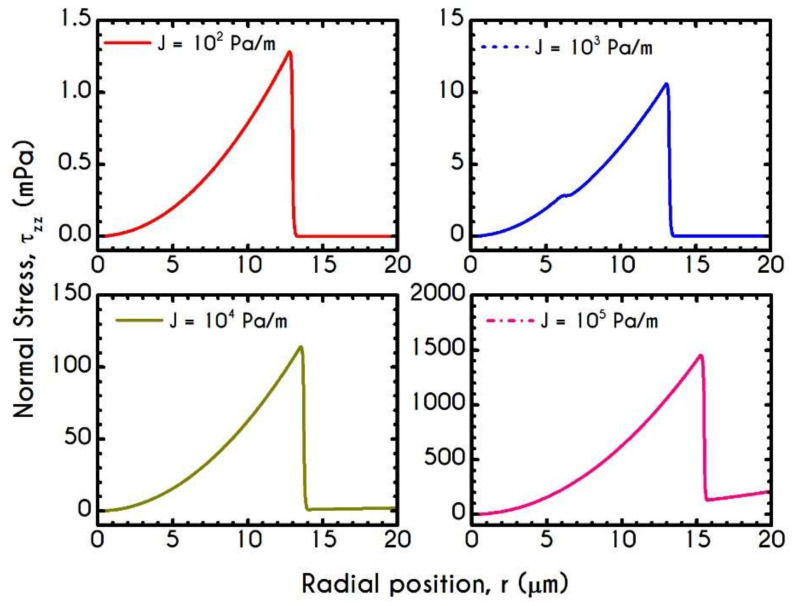
Normal viscoelastic stress $\tau_{\mathrm{ve},\mathrm{zz}}$ along the radial position for pressure gradient J equal to $10^{2}\;\mathrm{Pa}/m$, $10^{3}\;\mathrm{Pa}/m$, $10^{4}\;\mathrm{Pa}/m$, and $10^{5}\;\mathrm{Pa}/m$, for R = 20 μm and $H_{c}=0.45$.

**Figure 18:**
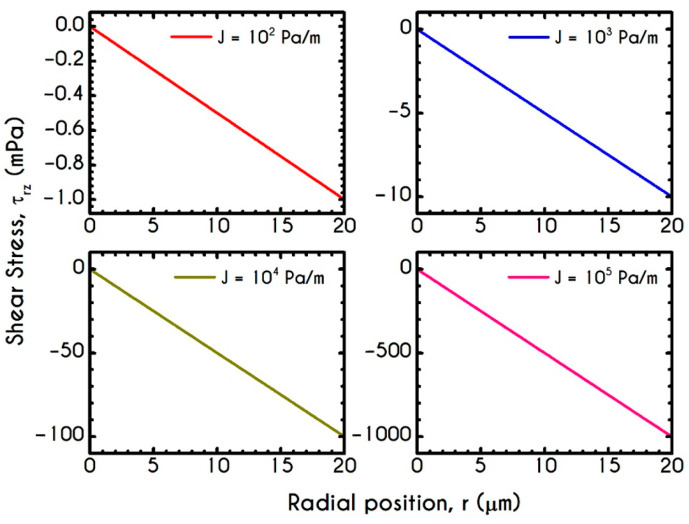
Shear viscoelastic stress $\tau_{\mathrm{ve},\mathrm{rz}}$ along the radial position for different pressure gradients J equal to $10^{2}\;\mathrm{Pa}/m$, $10^{3}\;\mathrm{Pa}/m$, $10^{4}\;\mathrm{Pa}/m$, and $10^{5}\;\mathrm{Pa}/m$ for R = 20 μm and $H_{c}=0.45$.

Figure 17 shows the normal viscoelastic stress distributions for different pressure gradients. An increase in J from $10^{2}\;\mathrm{Pa}/m$ to $10^{5}\;\mathrm{Pa}/m$ causes an increase in stress magnitude in both core and plasma regions. Regarding the RBC-rich central region, $\tau_{\mathrm{ve},\mathrm{zz}}$ demonstrates a continuous non-linear increase as the distance from the center is increased too. In the plasma phase, we can observe that the developed stress is quite insignificant, but when blood flows at higher velocities, as those depicted by the case with $J=\;10^{5}\;\mathrm{Pa}/m$, our model predicts an observable contribution of normal stress in the plasma phase. In this case, the rheological behavior is quite reasonable as we do not expect a considerable viscoelastic contribution from plasma. However, further increase in J yields a considerable normal stress distribution along the plasma layer, which is comparable to that predicted for the RBCs. This observation is in excellent agreement with the findings in the work of Varchanis et al. [60]. They predicted a pronounced normal stress, caused by the extension of plasma proteins, especially in high shear rates. This extra elastic contribution to the rheological response of whole blood may have a significant impact on the red blood cell deformation and interaction when flowing in microtubes. The effect of the pressure gradient on the viscoelastic shear stress distribution is presented in Figure 18. As expected, $\tau_{\mathrm{ve},\mathrm{rz}}$ implies a linear dependence on the radial position r along the tube as it is the total stress, the magnitude of which is strongly associated with the imposed pressure gradient.

**Figure 19:**
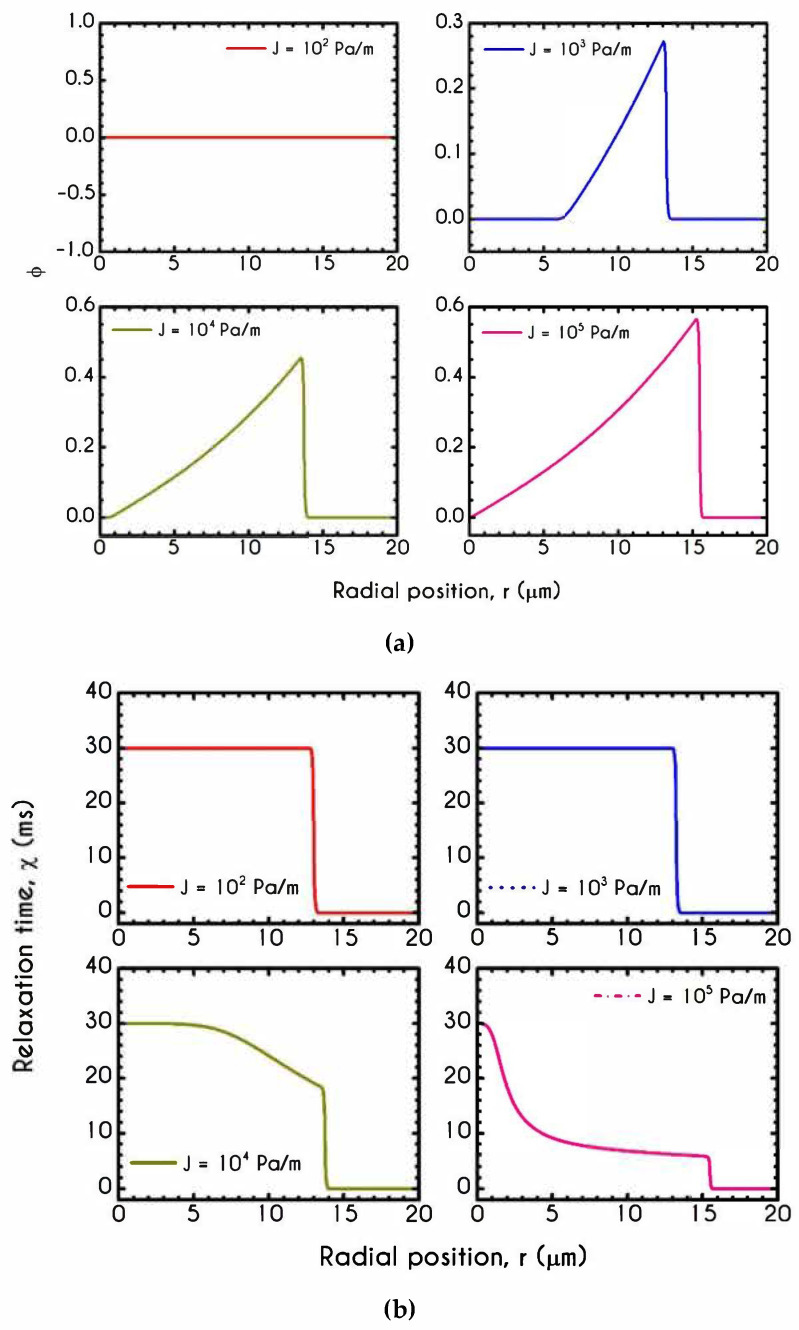
(**a**) Parameter φ along the radial position and (**b**) Relaxation time χ for different pressure gradients J equal to $10^{2}\;\mathrm{Pa}/m$, $10^{3}\;\mathrm{Pa}/m$, $10^{4}\;\mathrm{Pa}/m$, and $10^{5}\;\mathrm{Pa}/m$ for R = 20 μm and $H_{c}=0.45$.

Figure 19a presents the φ parameter for different pressure gradients. It reveals that the pressure gradient has a significant impact on the state of blood regarding its yielded or unyielded regions. Since plasma does not demonstrate plasticity, φ has a dual role indicating both the unyielded blood and the absence of plasticity in the annulus region. For the lowest imposed J, the predicted behavior implies that φ=0 throughout the tube, i.e., unyielded blood. Increasing J to $J=10^{3}\;\mathrm{Pa}/m$, results in partial fluidization with an unyielded region up to 6 μm of the tube and a yielded region from this point up to the phase change location δ. On the contrary, the remaining two cases depict the total fluidization of blood. Although, the transition from the unyielded $\left(J\;=\;10^{2}\;\mathrm{Pa}/m\right)$ to completely yielded blood $\left(J\;=\;10^{4}\;\mathrm{Pa}/m\right)$ is quite steep, under intermediate conditions we would observe situations with partial fluidization. For the cases with $J=10^{3}\;\mathrm{Pa}/m$ to $J=10^{5}\;\mathrm{Pa}/m$, the region with zero φ is the plasma layer which is totally yielded demonstrating zero plasticity.

**Figure 20:**
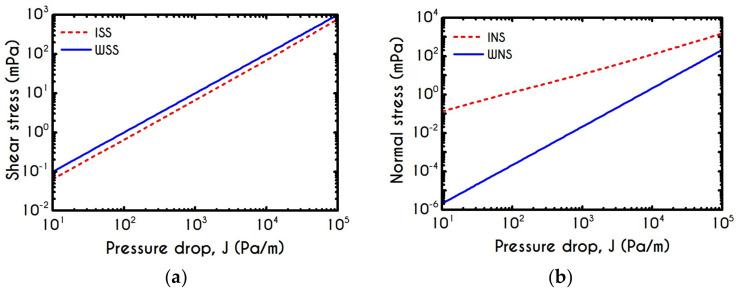
Prediction of (**a**) shear (ISS & WSS) and (**b**) normal (INS & WNS) interfacial and wall viscoelastic stresses as a function of the pressure gradient J for R = 20 μm and $H_{c}=0.45$.

The developed stresses are quite sensitive to pressure gradient imposition, as it is depicted in Figure 20a,b, which demonstrates the viscoelastic stresses at the blood/plasma interface and on the microtube wall. We can observe the monotonic variation of stress when the pressure gradient is ranging between 10 Pa/m to $10^{5}\;\mathrm{Pa}/m$ for a microtube with a radius equal to R = 20 μm and a constant core hematocrit equal to $H_{c}=0.45$. From Figure 20a, we can see that ISS is always lower than the WSS. In Figure 20b, INS attains higher values than WNS does, as it is expected. The viscoelastic contribution of blood is more significant than that of pure plasma, and therefore, we cannot observe any excess of WNS.

**Figure 21:**
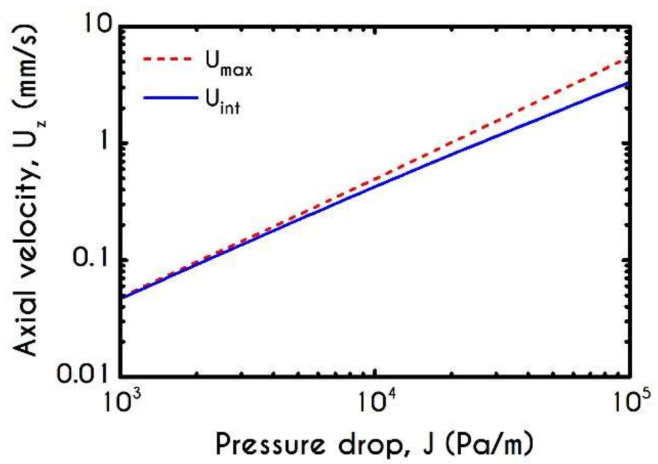
Maximum $U_{\max}$ and interfacial velocity $U_{\mathrm{int}}$ as a function of pressure gradient J for R = 20 μm and $H_{c}=0.45$.

Figure 21 demonstrates the prediction for the interfacial velocity $U_{\mathrm{int}}$ and the maximum attainable velocity at the center of the tube $U_{\max}$ under the aforementioned rheological conditions. For low-pressure gradients up to $10^{3}\;\mathrm{Pa}/m$, the velocity profiles are almost plug and thus the $U_{\max}$ and the $U_{\mathrm{int}}$ acquire the same values. As the imposed pressure gradient increases, we observe that the two curves begin to deviate from each other up to $J=\;10^{5}\;\mathrm{Pa}/m$ where the velocity profile tends to be parabolic, the predictions for the $U_{\max}$ and the $U_{\mathrm{int}}$ are 5.49 mm/s and 3.33 mm/s, respectively.

**Figure 22:**
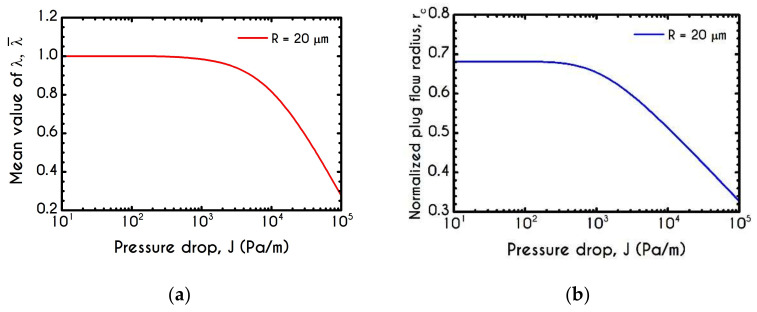
(**a**) Mean structure parameter $\bar{\lambda }$, (**b**) Normalized plug-flow radius $r_{c}$ as a function of the pressure gradient J for R = 20 μm and $H_{c}=0.45$.

In Figure 22a,b, we illustrate the mean microstructural configuration $\bar{\lambda }$ and the plug flow radius $r_{c}$, respectively. The flow conditions correspond to a pressure gradient range of 10–$10^{5}\;\mathrm{Pa}/m$, for microtube with a radius of R = 20 μm with a constant core hematocrit equal to $H_{c}=0.45$. Regarding Figure 22a, we can observe a quite expectable dependence of the mean structural variable on the pressure gradient J. For extremely low-pressure gradients, and hence low flow rates, the blood structure presents no change from the initial fully structured state up to a critical value of about $J=\;10^{3}\;\mathrm{Pa}/m$. From this point onward, the microstructure of blood starts to disintegrate, and $\bar{\lambda }$ experiences an abrupt and continuous reduction from 1 to 0.25. From Figure 22b, we can observe that the prediction of the normalized plug-flow radius $r_{c}$ as a function of the imposed pressure gradient is quantitatively similar to that presented for the mean value of λ (Figure 22a). Particularly, we observe two distinct responses of blood regarding the normalized region for which the blood velocity presents a plug profile. Initially, for low-pressure gradients, the velocity profile is plug throughout the core region, and hence the $r_{c}$ is constant and about 0.68. As the pressure gradient increases from $J\;=\;10^{3}\;\mathrm{Pa}/m$ to $J\;=\;10^{5}\;\mathrm{Pa}/m$ the velocity profile gradually obtains a more parabolic pattern, and thus the plug flow region is reduced dramatically to $r_{c}=0.32,$ which corresponds to the extreme pressure gradient value of our simulations.

## 5. Conclusions

This work presents steady-state predictions of blood flow in microtubes incorporating a two-layer fluid model consisting of an outer annulus filled with plasma and an inner RBC-rich core. Two constitutive formulations are employed, a thixotropic elasto-visco-plastic (TEVP) model for the blood core and a viscoelastic (linear-PTT) for the plasma, offering a holistic formulation for the accurate two-phase rheological behavior of blood/plasma in microtubes. The blood model was parameterized by Giannokostas et al. [4], using the hemorheological data of donors with 45% core hematocrit [55]. This is the first time that a TEVP model is validated in non-rheometric flows or flows with spatial variation of the velocity and the stress fields. The model is accompanied with CFL existence via empirical expressions of Pries and Secomb [92], and we have proposed a new simplified form (Equation (20)) compared to that provided by Pries et al. [74] for the description of the apparent viscosity. We present the predictions of the new analytical relationship of the CFL thickness with the radius of the tube. Comparison with experiments enables us to claim that our formulation constitutes a consistent model capable of successfully addressing complex blood flow behavior in narrow microtubes. In particular, we compared our predictions with experimental data of velocity profiles [76], cell-free layer thickness [5,77,78], as well as wall shear-stress measurements [79], obtaining an excellent agreement. We offered a thorough parametric analysis examining the impact of the microtube diameter as well as the pressure gradient on velocity profiles, normal and shear viscoelastic stresses, and the structural state of blood. Among the findings, we can summarize those that are of greater importance. Elasticity is found to have a significant impact on both RBCs and plasma phases. The normal stress field would be important in flows with a high flow rate or where extensional phenomena are quite intense. Additionally, elasticity provoked considerable discrepancies between TEVP and Casson predictions regarding the flow rate and the interfacial velocity. It was shown that the radius of the tube and the pressure gradient have a significant impact on the configuration of the internal structure of blood. For narrow microtubes, the aggregation was intense, the blood state was almost fully structured, leading to a blunted velocity profile. In addition, the relaxation time of the blood was also affected by the radius of the tube depicting higher values for narrower microtubes. As the location of the CFL is coincident with the region of the highest shear rate within the flow, the presence of such a layer was found to reduce the apparent viscosity significantly. Our model encompassed migration through a hemodynamical mathematical description, which was verified by experimental evidence and hence was able to predict the variation in local hematocrits accurately. For a small microtube, the model predictions implied a considerable reduction in discharged hematocrit due to migration effects, which in some cases is up to 70% and was proved to be invariant with the pressure gradient. The pressure gradient had a significant impact on the velocity profile and the mean microstructure of blood. We show a transition from totally unyielded to fully yielded blood with intermediate states of partial fluidization. The size of each region is linked with the imposed pressure gradient. The reduction of the blood structure λ within a reasonable range of pressure gradient was almost 80%.

## Figures and Tables

**Table 1:** Fitted rheological parameters of the TEVP model on steady and transient experimental hemorheological data reported in [55].

Symbol	Name of Variable	Units	Values
G	Elastic modulus	Pa	0.382
$\eta_{0}$	Plastic viscosity	Pa·s	0.012
$\tau_{y}$	Yield stress	Pa	0.0035
$\varepsilon_{\mathrm{PTT}}$	Extensional viscosity limiter	−	0.001
$k_{1}$	Brownian collisions scale	$s^{-1}$	0.0918
$k_{2}$	Shearing scale	$s^{n_{1}-1}$	7.249
$k_{3}$	Breakdown scale	$s^{n_{2}-1}$	6974.9
$n_{1}$	-	−	3.03
$n_{2}$	-	−	4.068
$n_{3}$	-	−	3.03
$m_{1}$	Plastic viscosity thixotropic scale	−	0.701

**Table 2:** Fitted rheological parameters of the Casson model on steady experimental data reported in [55].

Symbol	Name of Variable	Units	Values
$\tau_{y}$	Yield Stress	Pa	0.0033
μ	Viscosity	Pa·s	0.00389

**Table 3:** Parameters exported by the non-linear fitting of the linear-PTT model to plasma data [60].

Parameter	Name of Variable	Units	Value
$\lambda_{\mathrm{pl}}$	Relaxation time	s	$12.67\times 10^{-5}$
$\varepsilon_{\mathrm{PTT},\mathrm{pl}}$	Extensional viscosity limiter	−	$5\times 10^{-5}$
$\eta_{\mathrm{pl}}$	Plasma viscosity	Pa ·s	$1.9\times 10^{-3}$

**Table 4:** Model predictions for CFL thickness (w), flow rate (Q), Interfacial Normal Stress (INS), Interfacial Shear Stresses (ISS), Wall Shear Stress (WSS), and Wall Normal Stress (WNS) for microtubes with a radius equal to 20 μm and 35 μm.

Parameter	Name of Variable	Units	Values for R = 20 μm	Values for R = 35 μm
w	CFL thickness	μm	3.3	3.85
ISS	Interfacial shear stress	Pa	1.4	0.67
INS	Interfacial normal stress	Pa	0.45	0.23
WSS	Wall shear stress	Pa	1.67	1.04
WNS	Wall normal stress	Pa	3.23	2.38
Q	Flow rate	${\mathrm{mm}}^{3}/s$	$2.59\times 10^{-3}$	$4.67\times 10^{-4}$

**Table 5:** Coefficients of Equation (23) for ISS, WSS, INS, and WNS for $U_{\mathrm{mean}}\;=\;1\;\mathrm{mm}/s$ and $H_{c}=0.45$.

Parameters	Units	Values for ISS	Values for WSS	Values for INS	Values for WNS
$a_{n}$	Pa	−0.303	−0.417	4.75	0.135
$b_{n}$	Pa/μm	0.0043	0.00697	−0.022	−0.0128
$c_{n}$	$\mathrm{Pa}/{\mu m}^{2}$	$-2.55\times 10^{-5}$	$-4.43\times 10^{-5}$	$6.42\times 10^{-5}$	$5.37\times 10^{-4}$

**Table 6:** Coefficients of Equation (24) for the CFL thickness evaluation.

Parameter	Units	Values
$w_{1}$	μm	5.602
$w_{2}$	−	$2.43\times 10^{-2}$
$w_{3}$	1/μm	$-8.48\times 10^{-3}$
$w_{4}$	$1/{\mu m}^{2}$	$3.56\times 10^{-4}$

**Figure A1:**
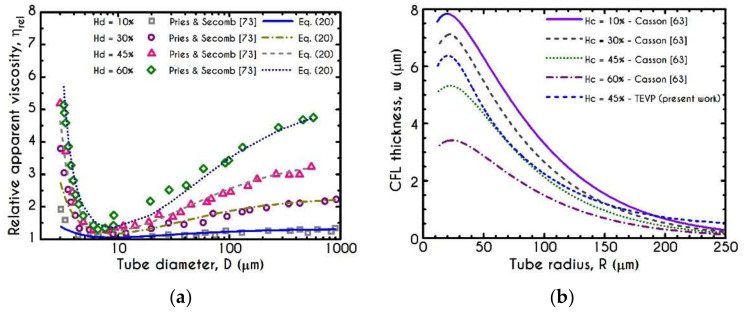
(**a**) The predictions of our proposed expression (Equation (20)) for relative apparent viscosity against experimental data reported by Pries and Secomb [73]. (**b**) Cell-free layer variation with respect to the tube radius and different values of core hematocrit based on Equation (20) for two different constitutive models applied in the core-annular flow problem described in Section 2.

**Figure A2:**
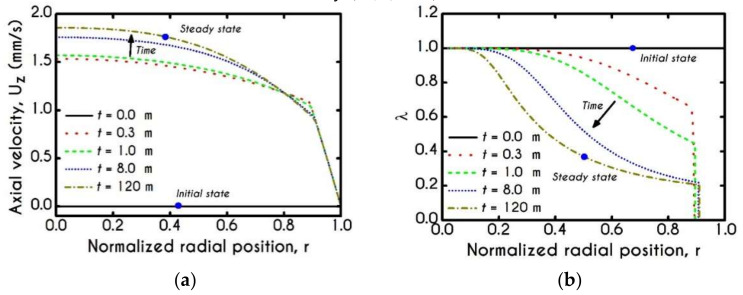
Time evolution of (**a**) axial velocity $U_{z}$, and (**b**) structure parameter λ from fully structured state to steady-state.

## Data Availability

The data presented in this study are available on request from the corresponding author.

## Appendix A

The reduced form of the z-component of Equation (1) in cylindrical coordinates is given by

(A1) ρ1 ∂Uz1∂t =− ∂P∂z+ 1r ∂∂rr τve,rz1  r ∈ 0,δ

(A2) ρ2 ∂Uz2∂t =− ∂P∂z+ 1r ∂∂rr τve,rz2  r ∈ δ,R

where the first equation refers to the blood-core region, while the second one to the blood plasma in the annulus. $U_{z}{}^{\left(1\right)}$ and $U_{z}{}^{\left(2\right)}$ are the velocities of the core region and plasma in the z-direction, respectively, $\tau_{\mathrm{ve},\mathrm{rz}}{}^{\left(1\right)}$ and $\tau_{\mathrm{ve},\mathrm{rz}}{}^{\left(2\right)}$ are the shear components of the viscoelastic stress tensor in core and plasma phases, respectively, $\frac{\partial P}{\partial z}$ is the pressure drop in the z-direction, and δ is the location of the blood/plasma interface. $\rho^{\left(1\right)}$ and $\rho^{\left(2\right)}$ are the densities of the blood and plasma equal to $1060\;\mathrm{Kg}/m^{3}$ and $1025\;\mathrm{Kg}/m^{3}$, respectively. It must be pointed out that a two-phase representation is mandatory not only for the distinct rheological description of each phase but also for the prediction of the interface position and, consequently, the computation of the cell-free layer (CFL) thickness w=R−δ. We also impose two boundary conditions for the velocity, a zero gradient at the center of the tube and a no-slip condition on the wall given by

(A3) $${\left.\frac{\partial U_{z}{}^{\left(1\right)}}{\partial r}\right|}_{r=0}\;=\;0$$

(A4) $${\left.U_{z}{}^{\left(2\right)}\right|}_{r=R}=\;0$$

At the blood/plasma interface, we impose continuity conditions for both stress and velocity as

(A5) $${\left.\tau_{\mathrm{ve},\mathrm{rz}}{}^{\left(1\right)}\right|}_{r=\delta }\;=\;{\left.\tau_{\mathrm{ve},\mathrm{rz}}{}^{\left(2\right)}\right|}_{r=\delta }$$

(A6) $${\left.U_{z}{}^{\left(1\right)}\right|}_{r=\delta }=\;{\left.U_{z}{}^{\left(2\right)}\right|}_{r=\delta }$$

As we have a moving boundary problem, due to the unknown location of the blood/plasma phase, the equations are solved in a transformed computational domain with fixed boundaries, and the solution is transformed back to the physical domain. The reduced one-dimensional forms of the constitutive law for the shear $\left(\tau_{\mathrm{ve},\mathrm{rz}}{}^{\left(1\right)}\right)$ and normal $\left(\tau_{\mathrm{ve},\mathrm{zz}}{}^{\left(1\right)}\right)$ components of the stress tensor in the core are given by

(A7) $$\frac{1}{2\;G}\frac{\partial \tau_{\mathrm{ve},\mathrm{rz}}{}^{\left(1\right)}}{\partial t}+\;f\left[\mathrm{tr}\left({\underline{\underline{\tau }}}_{\mathrm{ve}}^{\left(1\right)}\right)\right]\max\left(0,\frac{\left|{\underline{\underline{\tau }}}_{\mathrm{ve}}^{\left(1\right)}\right|-{\;\tau }_{y}}{2{\;\eta }_{t}\;\left|{\underline{\underline{\tau }}}_{\mathrm{ve}}^{\left(1\right)}\right|}\right)\tau_{\mathrm{ve},\mathrm{rz}}{}^{\left(1\right)}=\;\frac{\partial U_{z}{}^{\left(1\right)}}{\partial r}$$

(A8) $$\frac{1}{2\;G}\left(\frac{\partial \tau_{\mathrm{ve},\mathrm{zz}}{}^{\left(1\right)}}{\partial t}-2\frac{\partial U_{z}{}^{\left(1\right)}}{\partial r}\partial \tau_{\mathrm{ve},\mathrm{rz}}{}^{\left(1\right)}\right)+\;f\left[\mathrm{tr}\left({\underline{\underline{\tau }}}_{\mathrm{ve}}^{\left(1\right)}\right)\right]\max\left(0,\frac{\left|{\underline{\underline{\tau }}}_{\mathrm{ve}}^{\left(1\right)}\right|-{\;\tau }_{y}}{2{\;\eta }_{t}\;\left|{\underline{\underline{\tau }}}_{\mathrm{ve}}^{\left(1\right)}\right|}\right)\tau_{\mathrm{ve},\mathrm{zz}}{}^{\left(1\right)}=\;0\;$$

Accordingly, the components of the polymeric stress tensor for the plasma phase are given by

(A9) $$\tau_{\mathrm{ve},\mathrm{rz}}{}^{\left(2\right)}=\frac{\eta_{\mathrm{pl}}\;}{1+\;\frac{\varepsilon_{\mathrm{PTT},\mathrm{pl}\;}\lambda_{\mathrm{pl}}}{\eta_{\mathrm{pl}}}{\;\tau }_{\mathrm{ve},\mathrm{zz}}{}^{\left(2\right)}}\frac{\partial U_{z}{}^{\left(2\right)}}{\partial r}$$

(A10) $$\tau_{\mathrm{ve},\mathrm{zz}}{}^{\left(2\right)}=2\frac{\lambda_{\mathrm{pl}}}{\eta_{\mathrm{pl}}}{\left(\tau_{\mathrm{ve},\mathrm{rz}}{}^{\left(2\right)}\right)}^{2}$$

## Appendix B

The original form of the empirical description of the in vitro apparent viscosity experiments has been given by Secomb & Pries [94]. They assembled results from several studies, and they developed algebraic relationships to describe the dependence of the relative apparent viscosity $\eta_{\mathrm{rel}}$ (the ratio of the apparent viscosity to the suspending medium viscosity) on tube diameter and discharged hematocrit as

(A11) $$\eta_{0.45}\left(D\right)=3.2+220\exp\left(\;-1.3\;D\right)-2.44\;\exp\left(-0.06D^{0.645}\right)$$

(A12) $$C\left(D\right)=\left(0.8+\exp\left(-0.075D\right)\right)\;\left(-1+\frac{1}{1+10^{-11}D^{12}}\right)+\frac{1}{1+10^{-11}D^{12}}$$

(A13) $$\eta_{\mathrm{rel}}\left(H_{d},D\right)=1+\left(\eta_{0.45}\left(D\right)-1\right)\;\left(\frac{{\left(1-H_{d}\right)}^{C\left(D\right)}-1}{{\left(1-0.45\right)}^{C\left(D\right)}-1}\right)$$

where D is the diameter of the tube in μm, and C a dimensionless function of D. The apparent viscosity for a typical discharged hematocrit in humans $H_{d}=0.45,{\;\eta }_{0.45}$, is given by Equation (A11). The variable C (Equation (A12)) describes the type of dependence of the relative viscosity on the hematocrit, which is approximately linear for diameters up to about 8 μm, but shows a highly nonlinear dependence at large diameters. Finally, the expression of relative apparent viscosity $\eta_{\mathrm{rel}}$ is given by Equation (A13) based on the aforementioned quantities. This set of equations is much more complex compared to our proposed expression for the relative apparent viscosity given by Equation (20). Additionally, the dependence of parameter C on $D^{12}$ is quite unreliable, and prone to large errors. The prediction of the relative apparent viscosity with respect to the tube diameter for different values of discharge hematocrit, according to our equation, is illustrated in Figure A1a along with the corresponding experimental data given by Pries and Secomb [3]. The trend of the apparent viscosity to decrease with reducing the tube diameter continues down to about 7 μm. At smaller diameters, the relative apparent viscosity rises rapidly as the diameter approaches a critical minimum diameter, which is about 3 μm. Although highly deformable, RBCs are subject to constraints of constant volume and almost constant surface area. These constraints prevent passage of intact cells through tubes narrower than this critical diameter. As diameter increases, the relative viscosity attains higher values which tend to asymptotically reach the relative viscosity given by Poiseuille’s law for a Newtonian fluid. Figure A1b illustrates the predictions of cell-free layer thickness in μm with respect to the tube diameter and for different imposed core hematocrits. As our analysis is valid only for a certain value of core hematocrit that of 45%, we provide the predictive capability of Equation (20) using the Casson model (Equation (12), Table 2) [63] for hematocrit values between 10% to 60%. The predictions are compatible with experimental indications exhibiting a continuous decrease as the diameter of the tube increases. At high cross-sections, the CFL thickness asymptotically reaches a small but finite constant value, depending on the imposed core hematocrit.

## Appendix C

Our model is inherently transient, in the sense that there exists an infinitude of steady states, which depend in a continuous manner on the initial conditions and state. Even if it is just the steady-state that is sought, one cannot simply discard the time derivatives; the steady-state must be obtained by a time integration with appropriate initial conditions. The initial conditions imposed for the solution of the governing equations are those corresponding to initially unyielded and unperturbed blood. To this end, we assume a zero stress field (${\left.\tau_{\mathrm{ve},\mathrm{rz}}{}^{\left(i\right)}\right|}_{t=0}={\left.\tau_{\mathrm{ve},\mathrm{zz}}{}^{\left(i\right)}\right|}_{t=0}=0$) and velocity field (${\left.U_{z}{}^{\left(i\right)}\right|}_{t=0}=0$), while the structure variable is assumed to be unity (${\left.\lambda \right|}_{t=0}=1$).

The evolution of the profile of the axial velocity $U_{z}$ is presented in Figure A2a for five-time instances. During the transient simulation, the velocity field is gradually formed from zero state to a blunted profile at a steady-state, as it is indicated by the respective symbols in the graph. As time increases, the velocity attains a higher flow rate with larger bluntness and lower CFL thickness. Figure A2b illustrates the evolution of the structure parameter λ for a radius equal to R = 50 μm and a pressure drop equal to $J=10^{4}\;\mathrm{Pa}/m$ to achieve steady-state conditions. The initial state for λ is ${\left.\lambda \right|}_{t=0}=1$ so that the blood is not affected by any prior history effect. The stresses develop rapidly, leading to a continuous breakdown of blood rouleaux and consequently promote a decrease of λ. Gradually, more of the material acquires values below unity until the steady-state is achieved, and a small region near the center of the tube still retains its original state of the fully structured configuration. The size of this region is clearly dependent on the imposed flow conditions and especially on the magnitude of the pressure gradient and, consequently, on the applied shear-rates. The higher the developed shear-rates, the narrower is the region of a fully structured state. Apparently, as shear-rate attains higher values, the bulk configuration of λ acquires lower values in the sense that the blood is getting softer, and the microstructural destruction is higher. Irrespective of the intensity of shear rate, λ never reaches 0, but asymptotes to zero for extreme values of the shear-rate. In other words, the blood never becomes fully unstructured. Additionally, the fully structured region is always present, no matter how shear-rate increases.
